# Structural Characterization and Anti-Gout Activity of a Novel Acidic *Sanghuangporus vaninii* Polysaccharide

**DOI:** 10.3390/molecules30173536

**Published:** 2025-08-29

**Authors:** Xu Zhang, Siyu An, Lanying Zhou, Chen Chen, Xue Yang

**Affiliations:** Jilin Province Product Quality Supervision and Inspection Institute, Changchun 130103, China

**Keywords:** structural characterization, anti-gout activity, *Sanghuangporus vaninii*, polysaccharide, monosodium urate

## Abstract

In this study, a novel polysaccharide (PSH) with potent anti-gout activity was extracted and separated from *Sanghuangporus vaninii* (*S. vaninii*). The structural characteristics of PSH were elucidated using analytical techniques. HPLC analysis revealed that PSH was a heteropolysaccharide with a molecular weight of 5.25 × 10^4^ Da. FT-IR, NMR, and GC-MS collectively demonstrated that PSH was a pyranose with both α and β configurations, primarily composed of Glcp-(1→, →4)-Glcp-(2→, →3)-Galp-(1→, and Araf-(1→ linkages. The cell viability confirmed the non-toxicity of PSH. CAT and SOD showed that compared with the model group, PSH significantly offset the oxidative damage induced by MSU (*p* < 0.01). The results from ROS and MDA mutually corroborated the antioxidant capacity of PSH. Furthermore, PSH effectively suppressed MSU-triggered inflammatory responses. The antioxidant and anti-inflammatory experiments provided evidence for the anti-gout efficacy of PSH. Collectively, these findings support the potential development of PSH as an anti-gout active substance.

## 1. Introduction

Gout is the most common inflammatory arthritis worldwide, characterized by severe pain during acute attacks. According to a study, 55.8 million people worldwide suffered from gout in 2020, an increase of 150.6% over the past decade. Additionally, the number of male gout patients was three times that of female patients. The total number of gout cases is expected to reach 95.8 million by 2050 [1]. And the incidence of gout is increasing every year. Acute symptoms of gout are caused by an inflammatory reaction triggered by monosodium urate (MSU) crystals. The uric acid would precipitate and crystallize if serum uric acid concentrations exceed the maximum solubility threshold in blood. The deposition of these urate crystals within joints or soft tissues induces the synthesis and elevation of the inflammatory cytokine interleukin-1β (1L-1β), which mediates the subsequent inflammatory response, leading to the characteristic pain and pathology of gout [2]. Consequently, inhibiting the inflammatory factor 1L-1β has emerged as an effective therapeutic strategy for the targeted treatment of gout. Furthermore, reactive oxygen species (ROS) are key molecules in complex cellular signaling networks that regulate a variety of intracellular pathways in vivo. The changes in ROS contents may lead to the development of gout [3,4]. Thus, the onset of gout is inextricably linked to cellular inflammation and oxidative stress. And inflammatory cytokines and ROS play pivotal roles in its treatment.

*Sanghuangporus vaninii* (*S. vaninii*) (*Basidiomycotina, Agaricomycetes, Hymenochaetaceae*) [5]) is a large perennial medicinal fungus primarily distributed across Asian countries [6]. *S. vaninii* has been utilized as a traditional Chinese herbal medicine for over two millennia [7]. However, the availability of wild *S. vaninii* fruiting bodies is declining annually, failing to meet the growing demand. Fortunately, *S. vaninii* has been successfully cultivated artificially in recent years. *S. vaninii* cultivated on wood logs for three years has demonstrated pharmacological effects comparable to wild counterparts [8]. Research indicates that *S. vaninii* contains polysaccharides [9], flavonoids [10], and other bioactive compounds [11], with a range of pharmacological properties, including anti-inflammatory [12], anti-tumor [13], and antioxidant activities [14]. The active substances extracted from *S. vaninii* are widely applied in pharmaceuticals and health supplements [15]. Clinical studies suggest that *S. vaninii* extracts exhibit promising therapeutic effects against gouty arthritis, attributed to their anti-inflammatory and antioxidant properties [16].

Polysaccharides are important water-soluble extracts of *S. vaninii*, known for their antioxidant, hypouricemic, immunomodulatory, and anti-inflammatory effects. *S. vaninii* polysaccharides can reverse elevated serum uric acid levels, and reduce serum and hepatic xanthine oxidase (XO) activity in hyperuricemic mice [17], positioning them as potential candidates for gout adjunctive therapies and hyperuricemia. The functional bioactivity of polysaccharides is intrinsically linked to their structural features. Nevertheless, detailed studies on the structural characteristics of *S. vaninii* polysaccharides and their specific anti-hyperuricemic activities remain unreported.

This study aimed to extract polysaccharides from *S. vaninii* and characterize their structural properties. The antioxidant and anti-inflammatory activities of these polysaccharides were evaluated using a hyperuricemic cell model based on RAW264.7 cells to assess their potential anti-gout activity. The research seeks to establish a theoretical basis for the rational development and high-value utilization of *S. vaninii* resources. The potential of *S. vaninii* polysaccharides is explored as functional ingredients in health foods targeting hyperuricemia.

## 2. Results

### 2.1. Extraction and Purification

**Figure 1:**
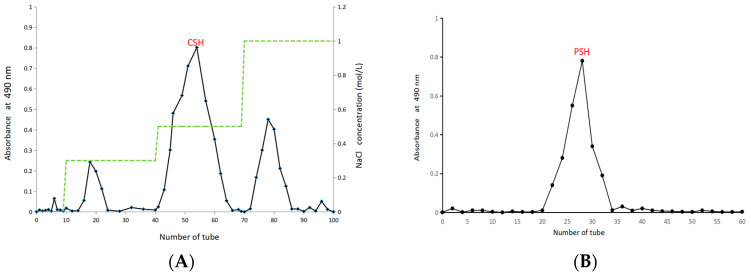
Chromatography of CSH on DEAE-52 (**A**); chromatography of PSH on Sephadex G-100 (**B**). The green dotted line in Figure 1A represents different concentrations of sodium chloride solution.

*S. vaninii* polysaccharide (CSH) was obtained via ultrasonic-assisted hot water extraction followed by ethanol precipitation, separated using a DEAE-52 cellulose weakly anionic exchanger. As shown in Figure 1A, different fractions of *S. vaninii* polysaccharides were eluted with NaCl solutions of varying concentrations, with the 0.5 mol/L NaCl eluate showing the highest yield. This fraction was selected for further purification on a Sephadex G-100 dextran gel column, yielding a single symmetrical peak in the elution profile (Figure 1B). The resulting purified polysaccharide was designated as PSH.

### 2.2. Chemical Composition Assay

**Figure 2:**
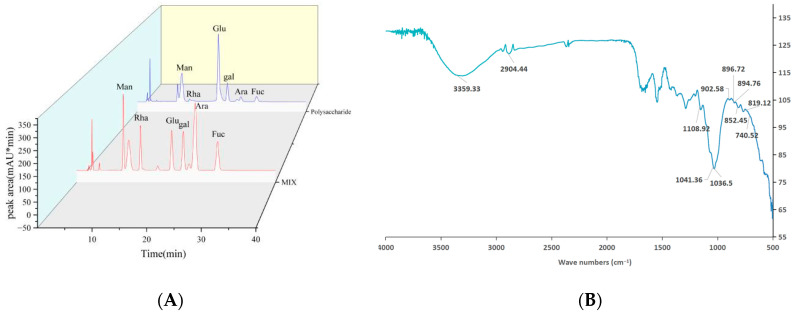
The monosaccharide composition of PSH (**A**); the infrared spectrum of PSH (**B**) (Table S1).

As shown in Table 1, the total sugar, protein, and uronic acid contents of PSH were 93.94%, 1.09%, and 17.05%, respectively. The molecular weight of the polysaccharide was determined by HPGPC. Using the average molecular weight as the ordinate and retention time as the abscissa, a standard curve was established: log(M_w_) = −0.3848 RT + 17.919 (R^2^ = 0.9995). Based on dextran standards of known molecular weights, the molecular weight of PSH was calculated to be 5.25 × 10^4^ Da. The monosaccharide composition of PSH was analyzed by high-performance liquid chromatography (HPLC). The results (Figure 2A) indicate that PSH contained Man, Rha, Glc, Gal, Ara, and Fuc with contents of 7.6%, 1.6%, 66.5%, 16.7%, 1.7%, and 5.9%, respectively.

### 2.3. FT-IR Assay

The functional groups and configuration of PSH were analyzed by FT-IR in the range of 400–4000 cm^−1^. As shown in Figure 2B, PSH displayed a broad and intense absorption peak around 3200–3600 cm^−1^, attributed to O-H stretching vibrations, characteristic of polysaccharides [18]. The absorption peaks at 1041 cm^−1^ and 1108 cm^−1^ corresponded to primary and secondary O-H bending vibrations, respectively. The absorption peaks at 2904 cm^−1^ and 1436 cm^−1^ were caused by methylene (-CH_2_-) vibrations. The absorption peaks at 894 cm^−1^ and 852 cm^−1^ indicated the presence of β- and α-configurations, respectively [19]. A typical absorption peak at 1036 cm^−1^ was assigned to pyran ring vibrations, confirming PSH as a pyranose. Vibrational peaks within the 740 cm^−1^-to-905 cm^−1^ range were characteristic of various pyranoses. Specifically, the peak at 904 cm^−1^ indicated β-D-glucopyranose, while the peak at 896 cm^−1^ was attributed to β-D-mannopyranose. The peaks at 902 cm^−1^ and 819 cm^−1^ suggested the presence of β-D-galactopyranose and α-D-galactopyranose, respectively, which was consistent with the results of the monosaccharide composition analysis.

### 2.4. Methylation Assay

Methylation analysis can resolve the glycosidic linkage types of the PSH glycosidic bond by GC-MS (Figure S1) and partially methylated alditol acetate (PMAA) library [20]. As shown in Table 2, the glycosidic linkages identified in PSH were as follows: →3,6)-Glcp-(2→ (12.4%), →4)-Glcp-(2→ (43.5%), Glcp-(1→ (11.2%), →3)-Galp-(1→ (16.4%), Araf-(1→ (2.1%), →3,5)-Araf-(2→ (1.7%), →4)-Fucp-(3→ (5.5%), →2)-Rhap-(1→ (2.1%), →6)-Manp-(1→ (5.1%). The main chain of PSH likely consisted of →4)-Glcp-(2→ and →3)-Galp-(1→ linkages, while Glcp-(1→ and Araf-(1→ serve as terminal sugars. The nearly equal ratio of terminal residues to branched residues indicated the complete methylation of the polysaccharide [21]. Furthermore, the methylation results largely agreed with the monosaccharide composition analysis.

### 2.5. NMR Assay

**Figure 3:**
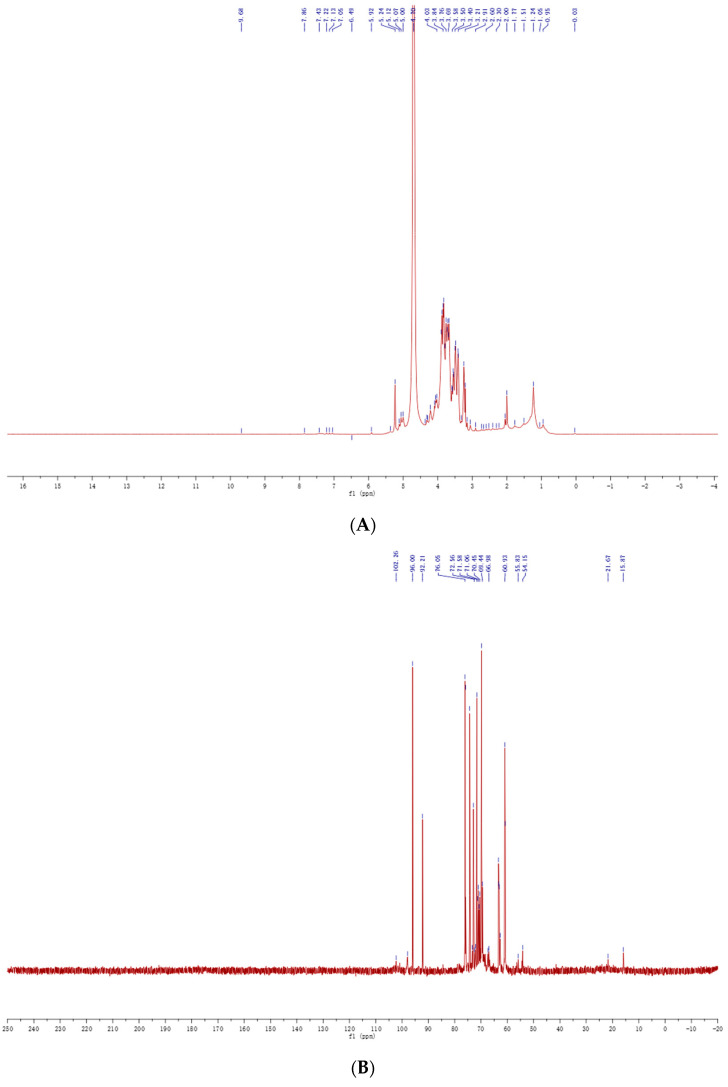
The ^1^H NMR (**A**) and ^13^C NMR (**B**) spectra of PSH.

To further elucidate the configuration and glycosidic linkages of PSH, ^1^H NMR and ^13^C NMR spectroscopy were performed. The ^1^H NMR spectrum of PSH (Figure 3A) exhibited characteristic chemical shift peaks at 1.2 ppm–1.3 ppm, corresponding to fucose residues. The absence of significant peaks in the 2.0 ppm–3.0 ppm region, which was characteristic of the protein, indicating efficient removal of the protein during purification. The dense cluster of multiple peaks within the 3.0 ppm–4.2 ppm region was typical for polysaccharides in ^1^H NMR. The signal peaks at 5.40 ppm confirmed PSH as a pyranose. Furthermore, anomeric proton signals appeared between 4.3 ppm and 5.3 ppm, demonstrating the presence of both α- and β-configurations [22], consistent with the FT-IR results. Nine anomeric proton signals were observed between 4.3 ppm and 5.8 ppm, aligning with the nine sugar residues identified by methylation analysis and signal splitting in this region was attributed to the D-configuration of sugars. The peak at 4.70 ppm originated from D_2_O. Due to the heterogeneous nature of the sample, only relative signal intensities are presented, without absolute proton number assignments or detailed coupling constants.

The ^13^C NMR spectrum of PSH (Figure 3B) displayed characteristic carbohydrate signals. Intense signals between 55 ppm and 65 ppm indicated the presence of exocyclic methylene carbon (-CH_2_-) signals. Chemical shift peaks in the regions 97 ppm–101 ppm and 103 ppm–105 ppm further verified the α- and β-configurations of D-pyranose in PSH. Resonances in the 90 ppm–105 ppm region were attributed to anomeric carbons, while peaks in the 60 ppm–85 ppm region corresponded to carbons in the sugar backbone (C-2 to C-6). Eight anomeric carbon signals of varying intensities appeared between 90 ppm and 110 ppm, indicating eight sugar residues. The discrepancy of one residue compared to the ^1^H NMR result was likely due to the lower sensitivity of ^13^C NMR for characterizing minor components. However, as expected for polysaccharide mixtures, because the carbon resonance strength was affected by relaxation time and the NOE effect, the ^13^C NMR spectrum of PSH was not integrated quantitatively.

### 2.6. Cell

#### 2.6.1. Cell Viability Assay

**Figure 4:**
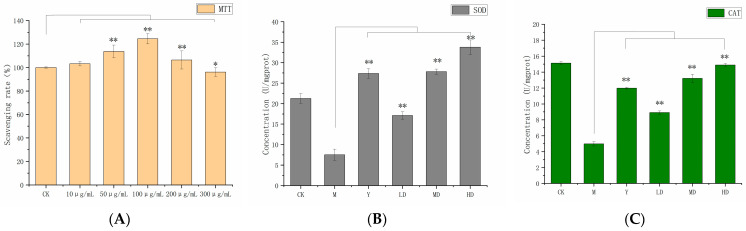
The viability of RAW 264.7 cells (**A**); SOD (**B**); and CAT (**C**) levels in RAW 264.7 cells. The control group (CK), the model group (M), the positive control (Y), and high (HD), medium (MD), and low (LD) experimental groups. Data are presented as the mean ± SD (n = 3). ** indicates *p* < 0.01 and * indicates *p* < 0.05.

Cell viability was assessed to evaluate the potential cytotoxicity of *S. vaninii* polysaccharides. The effect of different concentrations of PSH on the viability of RAW 264.7 cells was determined using the MTT assay. As shown in Figure 4A, different PSH concentrations exhibited varying effects on cell growth. Compared with the control group, a PSH concentration higher than 50 μg/mL significantly enhanced the ability of cell proliferation (*p* < 0.05). Maximum cell viability (124%) was observed at 100 μg/mL. However, cell viability decreased to 102% at 200 μg/mL. Therefore, concentrations of 50 μg/mL, 100 μg/mL, and 200 μg/mL were selected as safe doses for subsequent bioactivity experiments.

#### 2.6.2. Antioxidant Activity Assay

As shown in Figure 4B,C, SOD and CAT levels were reduced in cells from the uric acid-added model group compared with the control group. With the increase in polysaccharide concentration, the levels of SOD and CAT increased in a dose-dependent manner. SOD and CAT levels in the low-concentration group (17.1 U/mgprot, 8.9 U/mgprot) were significantly higher than those in the model group (7.5 U/mgprot, 5.0 U/mgprot) (*p* < 0.01). The SOD and CAT high-concentration group (33.8 U/mgprot, 14.8 U/mgprot) showed higher levels than the positive control group (27.3 U/mgprot, 12.0 U/mgprot). Antioxidant enzyme experiments showed that PSH could exert antioxidant effects by promoting the production of SOD and CAT in RAW 264.7 cells.

**Figure 5:**
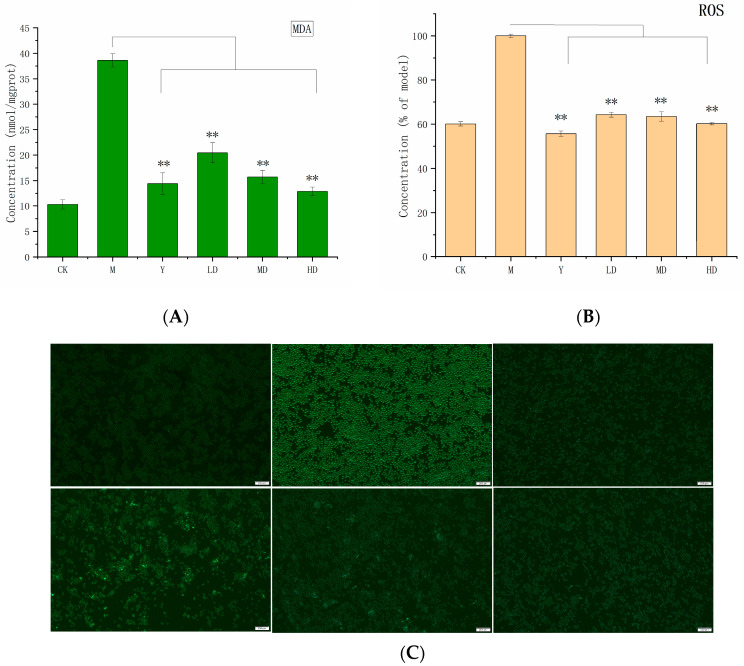
MDA levels in RAW 264.7 cells (**A**); ROS levels in RAW 264.7 cells (**B**); ROS fluorescence staining in RAW 264.7 cells (**C**). The control group (CK), the model group (M), the positive control (Y), and high (HD), medium (MD), and low (LD) experimental groups. Data of MDA and ROS are presented as the mean ± SD (n = 3). ** indicates *p* < 0.01, the model group was used as a control group.

ROS is a general term for peroxides that are related to oxygen metabolism, ROS contains oxygen free radicals and are easy to form free radicals in organisms, which can directly assess oxidative stress levels. The DCFH-DA probe was used to detect ROS levels in RAW 264.7 cells. As shown in Figure 5B,C, uric acid stimulation triggered substantial ROS production. The ROS content of the model group reached 30.1%, indicating the existence of oxidative stress. PSH experimental groups at different concentrations significantly depressed ROS secretion compared to the model group (*p* < 0.01). Furthermore, ROS levels in RAW 264.7 cells decreased progressively with increasing PSH concentration, demonstrating a dose-dependent effect. The ROS levels in the high-concentration group (60.1%) were comparable to those of the positive control group (57.8%).

MDA is a key parameter reflecting the antioxidant capacity of the body. MDA is an end product of lipid peroxidation and may serve as a biomarker of oxidative damage [23]. As shown in Figure 5A under monosodium urate MSU stimulation, RAW 264.7 cells produced substantial MDA. The MDA content in the model group (38.6 nmol/mgprot) was higher than in the blank group (10.3 nmol/mgprot), confirming successful establishment of the hyperuricemia model. The experimental groups exhibited the ability to inhibit MDA production. MDA levels showed a decreasing trend with an increasing PSH concentration. MDA content in experimental groups was significantly lower than that in the model group (*p* < 0.01). And the high-concentration experimental group (12.9 nmol/mgprot) even showed a lower MDA content than the positive control group.

#### 2.6.3. Anti-Inflammatory Activity

**Figure 6:**
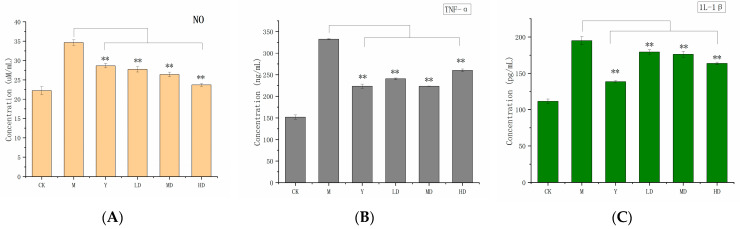
NO content in RAW 264.7 cells (**A**); TNF-α (**B**) and 1L-1β contents (**C**) in RAW 264.7 cells. The control group (CK), the model group (M), the positive control (Y), and high (HD), medium (MD), and low (LD) experimental groups. Data are presented as the mean ± SD (n = 3). ** indicates *p* < 0.01, the model group was used as a control group.

Nitric oxide is a key signaling molecule mediating inflammatory responses and involved in various physiological processes [24]. Excessive intracellular MSU crystals can trigger NO release, leading to inflammation. High intracellular NO levels can even damage the immune and nervous systems. Cellular NO content served as an indicator for evaluating the anti-inflammatory activity of PSH. The results (Figure 6A) showed that NO production in the model group was higher than in the blank group, confirming successful MSU modeling. With an increasing PSH concentration, the NO content in RAW 264.7 cells decreased, indicating that PSH inhibits cell inflammation caused by hyperuricemia. The NO contents in the medium-concentration experimental groups and high-concentration experimental groups were 26.4 µM/mL and 23.7 µM/mL, respectively, while that in the positive control group was 28.7 µM/mL. The anti-inflammatory capacity of the medium- and high-concentration groups was significantly greater than that of the model group (*p* < 0.01), which directly reflected the potent anti-gout activity of PSH.

TNF-α is a pleiotropic cytokine with a central role in inflammation, apoptosis, and immune system development [25]. Nevertheless, 1L-1β is a potent pro-inflammatory cytokine and a hallmark mediator of uric acid-induced inflammation [26]. As shown in Figure 6B,C, under the stimulation of high uric acid, RAW264.7 cells produced a large number of TNF-α and 1L-1β. The model group exhibited the TNF-α and 1L-1β contents of 332.7 ng/mL and 195.0 pg/mL, respectively. In the positive control group treated with colchicine, the secretion of TNF-α (223.4 ng/mL) and 1L-1β (138.6 pg/mL) in RAW 264.7 cells was reduced. The polysaccharide experimental groups exhibited significantly stronger inhibitory capacity than the model group (*p* < 0.01). The medium concentration group showed levels close to the ability of the positive control group to inhibit the production of TNF-α and 1L-1β. These results demonstrate that PSH effectively reduced the production of inflammatory cytokines, contributing to its anti-gout efficacy.

## 3. Discussion

In this study, we obtained acidic poplar-mushroom polysaccharides composed of Man, Rha, Glc, Gal, Ara, and Fuc. Notably, the glucose content exceeded 50%, and three or more monosaccharides were present, suggesting that PSH was a heteropolysaccharide with glucose as the main chain. This result was consistent with the previously reported monosaccharide composition of PFSV-2 [27]. Methylation combined with monosaccharide composition, Fourier transform infrared spectroscopy, and nuclear magnetic resonance analysis aids in the structural analysis of polysaccharides. The FT-IR and NMR results in this study mutually validated that poplar polysaccharides consist of α-type and β-type configurations, which was also consistent with previous research results [28]. Additionally, according to previous reports, the polysaccharide extracted from *S. vaninii* (SVP-a2) has a main chain composed of →6)-α-Galp-(1→ [29]. However, in this study, the main chain of poplar *S. vaninii* polysaccharide consists of →4)-Glcp-(2→ and →3)-Galp-(1→, which was inconsistent with previous results. This discrepancy might be attributed to differences in the extraction, separation, and purification methods used. However, the biological activity of polysaccharides stems from the synergistic effects of multiple structural factors [30,31,32]. Although this study conducted basic structural characterization of the *S. vaninii* polysaccharide, it did not adequately investigate information such as the conformation and tertiary structure of the polysaccharide.

*S. vaninii* has a long history as a medicinal fungus, and its medicinal safety has been well established [33,34]. Previous studies used CCk-8 to assess the cytotoxicity of *S. vaninii* polysaccharides on HT-22, Kupffer macrophages, and HEK293 cells. At the same time, the acute toxicity of different doses of polysaccharides on mice was evaluated. The results showed that *S. vaninii* polysaccharides had no toxic effects [35]. In this study, the safety of *S. vanini* polysaccharide was confirmed using the MTT assay. However, at polysaccharide concentrations exceeding 100 μg/mL, the ability to promote cell growth decreased, which may be due to high concentrations interfering with cell–culture medium interactions, leading to reduced activity.

Oxidative stress is closely associated with inflammatory responses. Enhancing antioxidant capacity can effectively reduce oxidative stress levels in gout patients while alleviating inflammatory responses [36]. There is clear evidence that excessive production of reactive oxygen species (ROS) may lead to cellular and tissue damage and trigger inflammatory responses [37]. Therefore, reducing ROS secretion can help counteract gout-related inflammation. In this study, compared with the model group, *S. vaninii* polysaccharides exhibited significant ROS inhibitory effects (*p* < 0.01), alleviating cell free radical damage induced by hyperuricemia. These results are supported by previous studies [38,39]. Additionally, due to the short half-life of ROS, the DCFH-DA quantification of ROS has certain uncertainties. We validated the ROS results using MDA to address this technical issue in this study. Furthermore, SOD and CAT play a key roles in the biological antioxidant system and serve as indicators of oxidative stress levels [40]. Previous studies have shown that polysaccharides can upregulate the secretion levels of SOD and CAT through the NRF2 signaling pathway, thereby exerting a protective effect against oxidative damage [41]. In our study, we also found that *S. vaninii* polysaccharides could reduce the production of SOD and CAT in the MSU oxidative stress model, thereby exerting an antioxidant effect, and this effect might be related to the NRF2 signaling pathway.

Gout is a disease caused by the deposition of monosodium urate (MSU) in joints, leading to joint inflammation. Therefore, reducing the deposition of MSU in joints is a key strategy for treating and alleviating gout [42]. Inflammatory cytokines, particularly IL-1β, are key mediators of gouty inflammation. Previous studies have exhibited that honeysuckle polysaccharides could exert anti-gout activity by downregulating the levels of inflammatory factors IL-1β, IL-6, and TNF-α in the serum of mice in a sodium urate-induced gouty arthritis model [43]. In this study, compared with the model group, the polysaccharide experimental group significantly downregulated the levels of inflammatory factors (IL-1β and TNF-α) in the MUS inflammatory cell model (*p* < 0.01). This results consistent with previous studies. Additionally, the NLRP3 inflammasome is the primary pathway through which MSU crystals trigger cellular inflammatory responses. Wang et al. authenticated that active extracts from *Cichorium intybus L.* could suppress gout by downregulating IL-1β release through the NF-κB and NLRP3 signaling pathways [44]. Using a MUS-induced oxidative and inflammatory cell model of RAW 264.7 cells, we demonstrated that poplar polysaccharides exhibit excellent protective effects against MSU-induced gout, achieved through antioxidant and anti-inflammatory mechanisms. These findings provide evidence supporting the potential of *S. vaninii* polysaccharides as active compounds for gout treatment. Unfortunately, due to funding and time constraints, this study was unable to investigate the mechanisms by which *S. vaninii* polysaccharides regulate gout or conduct in vivo experiments to validate their active effects, thereby limiting the scope of this research. Additionally, the relationship between polysaccharide structure and biological activity, as well as the underlying mechanisms, remain challenging areas for future research.

## 4. Materials and Methods

### 4.1. Materials and Reagents

The fruiting bodies of cultivated *S. vaninii* (3-year-old, artificial cultivation) were sourced from Yanbian Korean Autonomous Prefecture, Jilin Province, China, and provided by Jilin Sanghuang Biotechnology Group Co., Ltd. Glucose, galactose, mannose, rhamnose, fucose, and arabinose were purchased from Sigma-Aldrich Chemical Co. (Livonia, MI, USA). All other chemical reagents were of analytical grade and obtained from Beijing Chemical Works (Beijing, China). DEAE-52, Sephadex G-100, DMEM, fetal bovine serum (FBS), and methylthiazolyldiphenyl-tetrazolium bromide (MTT) were purchased from Solarbio Science & Technology Co., Ltd. (Beijing, China). Monosodium urate (MSU) and colchicine were obtained from Shanghai Yuanye Bio-Technology Co., Ltd. (Shanghai, China). The nitric oxide (NO) assay kit was purchased from Biyuntian Biotechnology Co., Ltd. (Shanghai, China). Enzyme-linked immunosorbent assay (ELISA) kits for cytokine detection were procured from Nanjing Jiancheng Bioengineering Institute (Nanjing, China).

### 4.2. Polysaccharide Extraction

Dried and powdered *S. vaninii* fruiting bodies (100 g) were extracted with 20 volumes (*v/v*) of distilled water under ultrasonication (60 °C, 2 h). The mixture was filtered, and the filtrate was concentrated to one-tenth of its original volume by rotary evaporator (R-215 BUCHI, Worcester, Switzerland). Four times the volume of anhydrous ethanol were added to the concentrate and stored at 4 °C for 24 h to precipitate polysaccharides. Precipitates were collected by centrifugation (X-30R Beckman, Brea, CA, USA) (8000 rpm, 10 min, 4 °C), dried in a 60 °C water bath to remove residual ethane, dissolved in a minimal volume of distilled water, extracted with 4 times the volume of the Sevag reagent (chloroform:n-butanol, 4:1, *v/v*), and vigorously shaken for 1 h. After phase separation in a separatory funnel, the precipitate was discarded. This process was repeated until no precipitate formed. The crude polysaccharide (CSH) was lyophilized at the cold trap temperature of −80 °C and vacuum degree of 15 Pa for 6 h using a freeze dryer (ZONE25 Labconco, Kansas, MO, USA).

### 4.3. Separation and Purification

The CSH was fully dissolved in distilled water, and centrifuged. The supernatant was applied to a DEAE-52 cellulose anion-exchange column. Elution was performed with distilled water, 0.3 mol/L, 0.5 mol/L, and 1.0 mol/L NaCl at 3 mL/min, collecting 10 mL fractions. The eluent in each tube was monitored using the phenol–sulfuric acid method. Elution profiles were generated by plotting the tube number as the horizontal axis against the absorbance of each eluent tube as the vertical axis. The elution solutions from each peak were combined, concentrated, dialyzed, and freeze-dried to obtain purified *S. vaninii* polysaccharides (PSHs). Polysaccharides were placed in sealed reagent bottles, and stored at 20 °C in a dryer.

### 4.4. Structural Characterization

#### 4.4.1. Chemical Composition Analysis

The content of total sugar, protein, and uronic acid was determined by the phenol sulfuric acid method [45], the Bradford method [46], and the sulfuric acidcarbazole method [47], respectively.

#### 4.4.2. Molecular Weight Analysis

Molecular weight analysis followed Zhang et al. [48] with slight modifications. The PSH was dissolved in distilled water to prepare a 2 mg/mL solution. High-performance gel permeation chromatography (HPGPC) on an Alliance 2695 (Waters, MA, USA) was performed on Ultrahydrogel 2000 (7.8 mm × 300 mm,12 µm) in series with a Ultrahydrogel 500 (7.8 mm × 300 mm, 10 µm) chromatographic column, and the column temperature was 40 °C. Distilled water was used as the mobile phase, and the refractive index detector (RID) was used as the detector. PSH molecular weight was calculated using a dextran standard curve.

#### 4.4.3. Monosaccharide Composition Analysis

Monosaccharide composition was analyzed by derivatization with 1-phenyl-3-methyl-5-pyrazolone (PMP) following Ji et al. [49] Polysaccharides were hydrolyzed with 10 mL of 2 mol/L trifluoroacetic acid (TFA), vortexed and mixed well, covered with a stopper, and placed in a high-temperature nitrogen-filled anaerobic oven at 120 °C for 2 h. The hydrolysate was then cooled to room temperature, dried with nitrogen, and dissolved in 1 mL of distilled water for later use. Then, 500 μL of 0.5 mol/L PMP and 500 μL of 0.5 mol/L NaOH were added to the solution and mixed well. The derivative liquid was incubated at 70 °C for 30 min. The derivative reactant was neutralized with 500 μL of 0.5 mol/L HCl and was extracted three times with chloroform (3 mL each). The aqueous phase was carefully collected and filtered by a 0.45 μm membrane. The polysaccharide was determined by HPLC (1260 Agilent, Santa Clara, CA, USA) equipped with a UV detector. Instrument conditions were as follows: chromatographic column: ZORBAX C18 (4.6 mm × 250 mm, 5 μm); mobile phase: acetonitrile (A): 0.1 mol/L ammonium acetate solution (B) = 20:80; column temperature: 30 °C; flow rate: 1.0 mL/min; detection wavelength: 254 nm; injection volume: 10 µL (Figure S2).

#### 4.4.4. Fourier Transform Infrared (FT-IR) Spectroscopy

First, 2 mg of the dried polysaccharide powder samples was weighed and thoroughly ground with 180 mg of KBr, pressed into pellets, and scanned at 4000–500 cm^−1^ using a RESTIGE-21 FT-IR spectrometer (Shimadzu, Kyoto, Japan).

#### 4.4.5. Nuclear Magnetic Resonance (NMR) Spectroscopy

PSH (20 mg) was dissolved in 0.5 mL of deuterium oxide (D_2_O) and transferred to a NMR tube. ^1^H NMR and ^13^C NMR spectra were recorded at 25 °C on a Bruker 600 MHz NMR spectrometer.

#### 4.4.6. Methylation

The polysaccharide samples were methylated and analyzed by GC-MS, referring to the method of Zhang et al. [50] with minor modifications. Briefly, 3 mg of polysaccharides were weighed and dissolved in 0.5 mL of DMSO. Then, 0.5 mL NaOH-DMSO suspension was added to the polysaccharide solution, stirred with a magnetic force for 1 h, slowly added with 1 mL CH_3_I, sealed with tin foil, and reacted at 4 °C for 0.5 h. Then, 2 mL of distilled water was added to terminate the reaction. The obtained reaction solution was dialyzed in running water for 24 h and freeze-dried to obtain dry polysaccharides. The dried polysaccharides were added with 2 mL TFA and reacted at 120 °C for 1 h. The mixed acid was removed with anhydrous ethanol to make the solution neutral to obtain methylated polysaccharide. Next, 1 mL of 35 mg/mL NaBH_4_ solution was added to the solution, stirred at room temperature for 12 h. The reaction was neutralized with approximately 100 μL of 50% glacial acetic acid, and the solution was passed through a strong acid cation-exchange resin. The eluate was lyophilized to obtain the reduced polysaccharides. Acetylation was carried out by adding 0.5 mL each of anhydrous pyridine and acetic anhydride to the reduced polysaccharides, followed by heating at 100 °C for 2 h. Acetic anhydride was removed with anhydrous ethanol, and the resulting acetylated sample was freeze-dried to obtain acetylated samples for gas chromatography–mass spectrometry (GC-MS) (7890B-5977 Agilent, Santa Clara, CA, USA) analysis. Methylated samples were separated on an Agilent Technologies DB-35 ms column (30 m × 0.25 mm × 0.25 μm). The conditions were as follows: carrier gas: helium; flow rate: 0.6 mL/min; injector temperature: 250 °C; injection volume: 1 μL (automatic injection, split ratio 1:5); mass spectrum conditions: EI source; ionization energy: 70 eV; ion source temperature: 250 °C; interface temperature 250 °C; oven temperature program: initial temperature 120 °C (held for 5 min), ramp to 250 °C at 2 °C/min (held for 20 min); total run time: 90 min.

### 4.5. Cell

#### 4.5.1. Cell Culture

RAW264.7 cells (catalog no. TIB-71, ATCC) were cultured in an H-DMEM medium (Hyclone, Marlborough, MA, USA) with 10% fetal bovine serum (Biological Industries, Kibbutz Beit, Israel) and 1% penicillin–streptomycin solution at 37 °C, 5% CO_2_, using passages 3–15 for all experiments.

#### 4.5.2. Cell Viability

Cells in the logarithmic growth phase were seeded into 96-well plates at a density of 1 × 10^5^ cells/well. After cell attachment, the medium was replaced with a complete medium containing varying concentrations of PSH. Following 24 h incubation, the medium was discarded, and 100 μL of MTT solution (0.5 mg/mL) was added to each well. The samples were shaken and incubated in a 37 C incubator for 4 h. After 4 h, the supernatant was removed, and 100 μL of Formazan was added. Plates were shaken in the dark for 10 min and cultured in 37 °C for 4 h. The absorbance was measured at 570 nm using an enzyme marker. The control group (CK) was not supplemented with samples. Cell viability was calculated using the formula as follows:

(1) $$\mathrm{Viability}\;(\%)=(A_{1}-A_{0}/A_{2}-A_{0})\times 100\%$$

where A_0_ = absorbance of distilled water, A_1_ = absorbance of the sample group, A_2_ = absorbance of the control group.

#### 4.5.3. Cell Experiment Groups and Treatments

Cells were cultured in 96-well plates at a density of 2 × 10^6^ cells and incubated at 37 °C for 24 h. Cells were then randomly assigned to different groups. In the model group (M), RAW264.7 cells were stimulated with 100 μg/mL MSU to create a hyperuricemic cell model. Colchicine can effectively treat gout, which is the most commonly used drug in the acute attack of gouty joints. The anti-gout activity of different concentrations of PSH was evaluated using colchicine (0.001 umol/L) + 100 μg/mL MSU as the positive control (Y). The 50, 100, 200 μg/mL PSH + 100 μg/mL MSU were used as high (HD), medium (MD), and low (LD) experimental groups, respectively. The control group (CK) was cultured without additives.

#### 4.5.4. Antioxidant Activity

The cells were cultured according to procedure 4.5.1, and lysed to extract intracellular proteins. The superoxide dismutase (SOD) activity, malondialdehyde (MDA) content, and catalase (CAT) activity were determined according to the instructions of relevant kits.

The ROS assay was performed according to the kit instructions. And cultured cells were treated as described in Section 4.5.1. After the culture was complete, 1 mL of 10 μmol/L DCFH-DA solution was added, and the cells were incubated in the dark at 37 °C for 20 min. The cells were washed three times with serum-free cell culture medium and then examined under a fluorescence microscope.

#### 4.5.5. Anti-Inflammatory Activity

Following treatment as described in Section 4.5.3, cell culture supernatants were collected and assayed for NO concentration using a reagent kit according to the manufacturer’s instructions. Absorbance was measured at 540 nm using an enzyme marker (Thermo Fisher, Waltham, MA, USA).

TNF-α and 1L-1β levels

The contents of tumor necrosis factor-α (TNF-α) and interleukin-1β (1L-1β) were detected by ELISA. RAW264.7 cells were cultured in 96-well plates at 2 × 10^6^ cells and incubated for 24 h. The cells were divided into a control group, a model group, a positive drug group, and low-, medium-, and high-dose groups of PSH. Cell supernatants were collected and assayed for TNF-α and 1L-1β contents using commercial ELISA kits according to the manufacturer’s instructions.

### 4.6. Statistical Analysis

All experimental data are presented as the mean ± standard deviation (SD) of three independent experiments. Origin 2021 and SPSS 19.0 software were used for the analysis of variance (ANOVA), Shapiro–Wilk, LSD, and Duncan’s multiple-range test. *p* < 0.05 was considered significant.

## 5. Conclusions

In this study, a polysaccharide (PSH) with potent anti-gout activity was isolated from *S. vaninii*. PSH was extracted, isolated, and purified from *S. vaninii*. Subsequently, its structural characteristics were elucidated using advanced analytical techniques. HPLC analysis revealed PSH to be a heteropolysaccharide with a molecular weight of 5.25 × 10^4^ Da. FT-IR, NMR, and GC-MS analyses collectively demonstrated that PSH is a pyranose with both α and β configurations, primarily composed of Glcp-(1→, →4)-Glcp-(2→, →3)-Galp-(1→, and Araf-(1→ linkages. Finally, cell viability assays confirmed the non-toxicity of PSH, which is the premise of bioactivity studies. CAT and SOD assays indicated that PSH significantly counteracted MSU-induced oxidative damage, which could effectively prevent oxidative stress. The results from ROS and MDA analyses mutually corroborated the antioxidant capacity of PSH. Furthermore, PSH significantly suppressed MSU-triggered inflammatory responses. Additionally, the antioxidant and anti-inflammatory experiments provided evidence for the anti-gout efficacy of PSH. This study offers a new pathway for product development within the industry of artificially cultivated *S. vaninii*.

## Figures and Tables

**Table 1:** Chemical composition date.

Fraction	Content	Monosaccharide	Content (%, Area)
Total sugar (%)	93.94 ± 3.55	Man	7.6
Protein (%)	1.09 ± 0.32	Rha	1.6
Uronic acid (%)	17.05 ± 2.71	Glc	66.5
		Gal	16.7
Molecular weight (Da)	5.25 × 10^4^	Ara	1.7
		Fuc	5.9

Note: Total sugar content was determined by the phenol–sulfuric acid method, protein content by the Bradford method, and uronic acid content by the *m*-hydroxydiphenyl method. Since these assays are based on different analytical principles and may partially overlap (e.g., uronic acids being a subclass of carbohydrates), the percentages cannot be simply added to 100%. The values represent results from independent assays rather than components of a proximate composition.

**Table 2:** Methylation analysis data.

Number	Methylation Product	Linkage Pattern	Area Ratio (%)
1	2,3,5,6-penta-O-acetyl-4-O-methyl-D-glucitol	→3,6)-Glcp-(2→	12.4
2	3,5,6-tetra-O-acetyl-2,4-di-O-methyl-D-glucitol	→4)-Glcp-(2→	43.5
3	5-di-O-acetyl-2,3,4,6-tetra-O-methyl-D-glucitol	Glcp-(1→	11.2
4	3,5-tri-O-acetyl-2,4,6-tri-O-methyl-D-galactitol/	→3)-Galp-(1→	16.4
5	5-di-O-acetyl-2,3,4-tri-O-methyl-D-arabinitol	Araf-(1→	2.1
6	2,3,4,5-penta-O-acetyl-D-arabinitol	→3,5)-Araf-(2→	1.7
7	3,4,5-tetra-O-acetyl-6-deoxy-2-O-methyl-L-galactitol	→4)-Fucp-(3→	5.5
8	2,5-tri-O-acetyl-6-deoxy-3,4-di-O-methyl-L-mannitol	→2)-Rhap-(1→	2.1
9	5,6-tri-O-acetyl-2-(acetylmethylamino)-2-deoxy-3,4-di-O-methyl-D-mannitol	→6)-Manp-(1→	5.1

## Data Availability

Data are contained within the article.

## Supplementary Materials

The following supporting information can be downloaded at: https://www.mdpi.com/article/10.3390/molecules30173536/s1, Figure S1. Total ion flux map of methylation test by gas chromatography mass spectrometry; Figure S2. Original spectrum of the monosaccharide composition. Standard reference chromatogram(A) and Polysaccharide chromatogram (B); Table S1. Original data of infrared spectrum.

## Abbreviations

The following abbreviations are used in this manuscript:

MSU	Monosodium urate
1L-1β	Interleukin-1β
ROS	Reactive oxygen species
XO	Xanthine oxidase
FBS	Fetal bovine serum
MTT	Methylthiazolyldiphenyl-tetrazolium
NO	Nitric oxide
ELISA	Enzyme-linked immunosorbent assay
HPGPC	High-performance gel permeation chromatography
RID	Refractive index detector
PMP	1-phenyl-3-methyl-5-pyrazolone
TFA	Trifluoroacetic acid
D_2_O	Deuterium oxide
GC-MS	Gas chromatography–mass spectrometry
SOD	Superoxide dismutase
MDA	Malondialdehyde
CAT	Catalase
TNF-α	Tumor necrosis factor-α
SD	Standard deviation
ANOVA	Analysis of variance
PMAA	Partially methylated alditol acetate
