# Supplementary material for: Structural Characterization and Anti-Gout Activity of a Novel Acidic Sanghuangporus vaninii Polysaccharide
Source: Molecules. 2025 Aug 29;30(17):3536. doi: 10.3390/molecules30173536 (PMC12429920; doi:10.3390/molecules30173536)
Supplement: Supplementary file 1 [file molecules-30-03536-s001.zip › molecules-3778262-supplementary.pdf]

Structural Characterization and Anti-Gout Activity of a Novel Acidic *Sanghuangporus vaninii* Polysaccharide

Supplementary Materials

Xu Zhang, Siyu An *, Lanying Zhou, Chen Chen and Xue Yang

Jilin Province Product Quality Supervision and Inspection Institute, Changchun 130103, China

* Correspondence: ansiyu@jlzjy.org

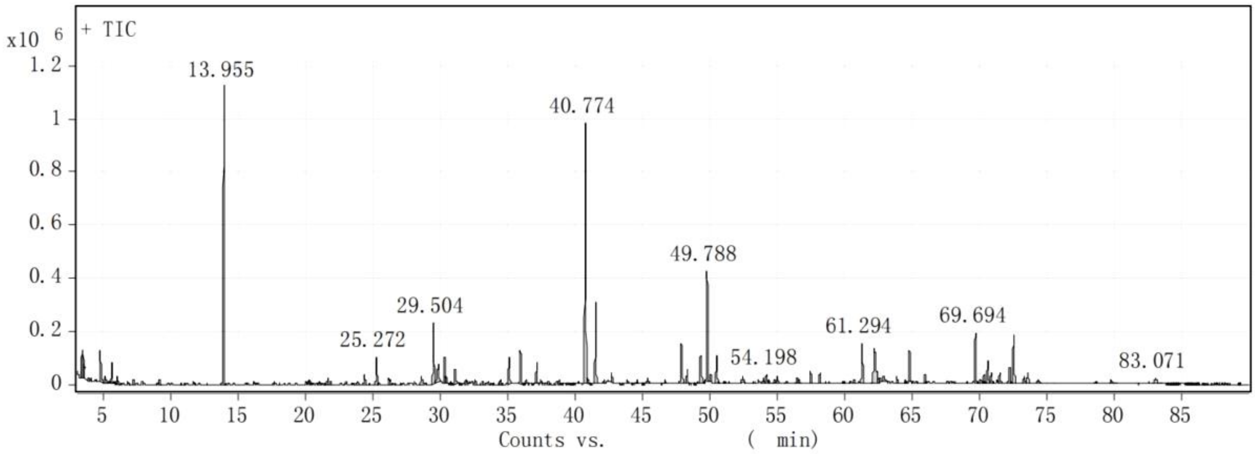

Figure S1. Total ion flux map of methylation test by gas chromatography mass spectrometry.

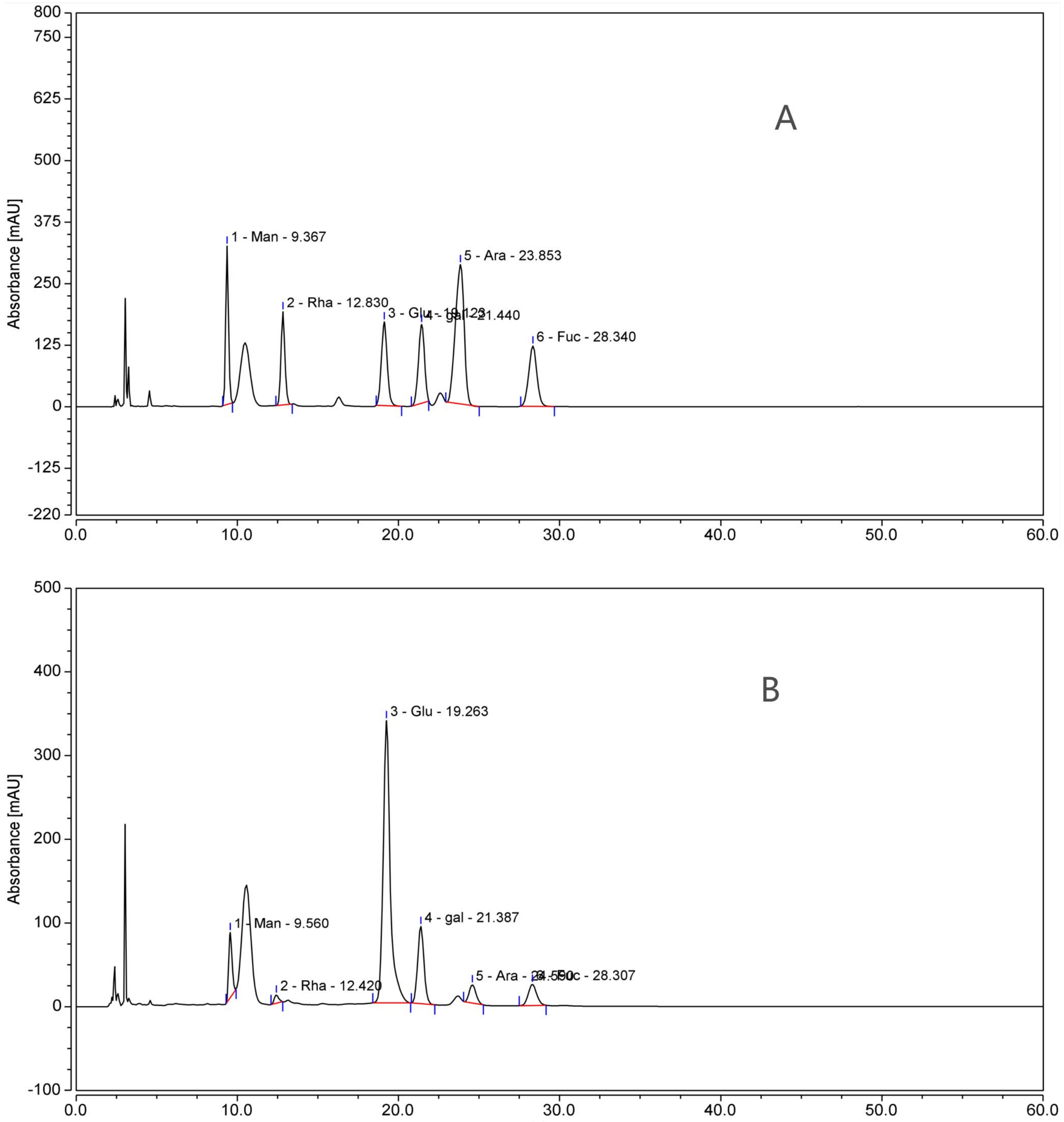

Figure S2. Original spectrum of the monosaccharide composition. Standard reference chromatogram(A) and Polysaccharide chromatogram(B).

Table S1. Original data of infrared spectrum

Number	Wave number (cm-1)	Transmissivity (%)
1	399.186	62.6298
2	401.115	55.4252
3	403.043	55.7279
4	404.971	66.6491
5	406.9	63.6482
6	408.828	57.1043
7	410.757	58.9797
8	412.685	58.1012
9	414.614	60.0382
10	416.542	64.7858
11	418.47	61.7365
12	420.399	58.4123
13	422.327	61.682
14	424.256	60.538
15	426.184	56.4523
16	428.113	61.1702
17	430.041	62.8879
18	431.97	56.5639
19	433.898	60.2133
20	435.826	68.06
21	437.755	67.3446
22	439.683	67.7265
23	441.612	66.9098
24	443.54	61.7699
25	445.469	60.203
26	447.397	60.9283
27	449.325	62.4166
28	451.254	62.8621
29	453.182	63.2882
30	455.111	64.5906
31	457.039	61.3919
32	458.968	58.5504
33	460.896	62.6398
34	462.824	66.9696
35	464.753	65.0706
36	466.681	64.2341
37	468.61	67.3599
38	470.538	64.7142
39	472.467	60.0103
40	474.395	62.0063
41	476.324	63.463
42	478.252	61.5452
43	480.18	61.3292
44	482.109	64.7901
45	484.037	66.682
46	485.966	62.5735
47	487.894	60.5477

48	489.823	65.0338
49	491.751	69.1042
50	493.679	68.6287
51	495.608	66.634
52	497.536	65.5252
53	499.465	67.4939
54	501.393	68.6348
55	503.322	63.5784
56	505.25	61.7334
57	507.178	66.0933
58	509.107	66.5441
59	511.035	65.4727
60	512.964	67.5509
61	514.892	66.8423
62	516.821	64.427
63	518.749	66.1963
64	520.678	69.2178
65	522.606	69.4145
66	524.534	68.9626
67	526.463	69.6065
68	528.391	70.0331
69	530.32	70.0333
70	532.248	70.8993
71	534.177	72.8586
72	536.105	74.6607
73	538.033	75.5956
74	539.962	76.1309
75	541.89	76.4467
76	543.819	76.7096
77	545.747	77.3444
78	547.676	77.4313
79	549.604	76.7808
80	551.533	76.984
81	553.461	77.6887
82	555.389	77.9082
83	557.318	77.9599
84	559.246	77.9141
85	561.175	77.9832
86	563.103	77.9872
87	565.032	77.7832
88	566.96	77.9102
89	568.888	77.9242
90	570.817	77.6638
91	572.745	77.7963
92	574.674	78.2959
93	576.602	78.5794
94	578.531	78.2007
95	580.459	77.9601
96	582.387	78.4212
97	584.316	79.2623
98	586.244	80.5223
99	588.173	81.4282

100	590.101	81.8011
101	592.03	82.3011
102	593.958	82.573
103	595.887	82.802
104	597.815	83.0718
105	599.743	82.986
106	601.672	82.8855
107	603.6	82.8233
108	605.529	82.773
109	607.457	82.8121
110	609.386	82.7988
111	611.314	82.8454
112	613.242	82.8905
113	615.171	82.8866
114	617.099	83.0248
115	619.028	83.3461
116	620.956	83.944
117	622.885	84.7228
118	624.813	85.3686
119	626.741	86.0629
120	628.67	86.8161
121	630.598	87.1363
122	632.527	87.1234
123	634.455	87.3074
124	636.384	87.7239
125	638.312	88.2205
126	640.241	88.6731
127	642.169	88.9702
128	644.097	89.3619
129	646.026	89.7819
130	647.954	90.0964
131	649.883	90.4487
132	651.811	90.7232
133	653.74	91.1301
134	655.668	91.5898
135	657.596	91.8822
136	659.525	92.3007
137	661.453	92.7994
138	663.382	93.506
139	665.31	94.1952
140	667.239	94.2206
141	669.167	94.2243
142	671.095	95.0628
143	673.024	95.5422
144	674.952	95.568
145	676.881	95.5924
146	678.809	95.7083
147	680.738	95.8461
148	682.666	95.9801
149	684.595	96.1492
150	686.523	96.4035
151	688.451	96.6463

152	690.38	96.9502
153	692.308	97.363
154	694.237	97.7486
155	696.165	98.0909
156	698.094	98.4169
157	700.022	98.6348
158	701.95	98.7221
159	703.879	98.8088
160	705.807	98.9411
161	707.736	99.0986
162	709.664	99.2314
163	711.593	99.3521
164	713.521	99.5637
165	715.45	99.7686
166	717.378	99.9201
167	719.306	100.067
168	721.235	100.146
169	723.163	100.286
170	725.092	100.546
171	727.02	100.736
172	728.949	100.807
173	730.877	100.85
174	732.805	100.893
175	734.734	100.967
176	736.662	101.105
177	738.591	101.249
178	740.519	101.316
179	742.448	101.345
180	744.376	101.416
181	746.304	101.517
182	748.233	101.598
183	750.161	101.627
184	752.09	101.6
185	754.018	101.544
186	755.947	101.439
187	757.875	101.266
188	759.804	101.104
189	761.732	100.973
190	763.66	100.829
191	765.589	100.735
192	767.517	100.708
193	769.446	100.706
194	771.374	100.781
195	773.303	100.948
196	775.231	101.174
197	777.159	101.421
198	779.088	101.646
199	781.016	101.871
200	782.945	102.109
201	784.873	102.383
202	786.802	102.699
203	788.73	102.966

204	790.658	103.142
205	792.587	103.253
206	794.515	103.27
207	796.444	103.124
208	798.372	102.924
209	800.301	102.814
210	802.229	102.638
211	804.158	102.383
212	806.086	102.269
213	808.014	102.241
214	809.943	102.237
215	811.871	102.272
216	813.8	102.244
217	815.728	102.228
218	817.657	102.325
219	819.585	102.458
220	821.513	102.673
221	823.442	102.941
222	825.37	103.115
223	827.299	103.328
224	829.227	103.628
225	831.156	103.821
226	833.084	103.93
227	835.012	104.034
228	836.941	104.102
229	838.869	104.192
230	840.798	104.252
231	842.726	104.177
232	844.655	104.045
233	846.583	103.919
234	848.512	103.866
235	850.44	103.914
236	852.368	104.006
237	854.297	104.151
238	856.225	104.341
239	858.154	104.562
240	860.082	104.75
241	862.011	104.821
242	863.939	104.911
243	865.867	105.087
244	867.796	105.225
245	869.724	105.282
246	871.653	105.362
247	873.581	105.487
248	875.51	105.557
249	877.438	105.567
250	879.367	105.517
251	881.295	105.408
252	883.223	105.307
253	885.152	105.188
254	887.08	105.047
255	889.009	104.981

256	890.937	104.958
257	892.866	104.935
258	894.794	105.034
259	896.722	105.181
260	898.651	105.201
261	900.579	105.232
262	902.508	105.335
263	904.436	105.414
264	906.365	105.461
265	908.293	105.459
266	910.221	105.43
267	912.15	105.348
268	914.078	105.189
269	916.007	105.054
270	917.935	104.906
271	919.864	104.706
272	921.792	104.562
273	923.721	104.448
274	925.649	104.275
275	927.577	104.08
276	929.506	103.885
277	931.434	103.672
278	933.363	103.449
279	935.291	103.172
280	937.22	102.826
281	939.148	102.513
282	941.076	102.213
283	943.005	101.856
284	944.933	101.504
285	946.862	101.167
286	948.79	100.813
287	950.719	100.409
288	952.647	99.9494
289	954.575	99.4915
290	956.504	98.9959
291	958.432	98.4178
292	960.361	97.8621
293	962.289	97.394
294	964.218	96.9416
295	966.146	96.4592
296	968.075	95.9978
297	970.003	95.5389
298	971.931	95.0223
299	973.86	94.4464
300	975.788	93.804
301	977.717	93.0566
302	979.645	92.1639
303	981.574	91.1885
304	983.502	90.1888
305	985.43	89.1666
306	987.359	88.2045
307	989.287	87.3258

308	991.216	86.5465
309	993.144	85.9654
310	995.073	85.551
311	997.001	85.1839
312	998.929	84.8317
313	1000.86	84.4737
314	1002.79	84.03
315	1004.71	83.5685
316	1006.64	83.2123
317	1008.57	82.9103
318	1010.5	82.6503
319	1012.43	82.4821
320	1014.36	82.376
321	1016.29	82.2459
322	1018.21	81.9737
323	1020.14	81.5192
324	1022.07	80.9984
325	1024	80.5684
326	1025.93	80.2627
327	1027.86	80.0492
328	1029.78	79.9312
329	1031.71	79.8742
330	1033.64	79.856
331	1035.57	79.9577
332	1037.5	80.2287
333	1039.43	80.6029
334	1041.36	81.0043
335	1043.28	81.431
336	1045.21	81.9706
337	1047.14	82.6086
338	1049.07	83.1839
339	1051	83.7199
340	1052.93	84.3134
341	1054.85	84.8513
342	1056.78	85.2112
343	1058.71	85.4356
344	1060.64	85.6343
345	1062.57	85.7901
346	1064.5	85.8568
347	1066.42	85.8915
348	1068.35	85.9157
349	1070.28	85.9495
350	1072.21	86.0664
351	1074.14	86.3126
352	1076.07	86.6918
353	1078	87.2088
354	1079.92	87.9231
355	1081.85	88.7863
356	1083.78	89.6971
357	1085.71	90.6889
358	1087.64	91.6897
359	1089.57	92.6044

360	1091.49	93.4498
361	1093.42	94.1567
362	1095.35	94.7179
363	1097.28	95.181
364	1099.21	95.5071
365	1101.14	95.7036
366	1103.07	95.838
367	1104.99	95.9708
368	1106.92	96.1624
369	1108.85	96.4759
370	1110.78	96.9018
371	1112.71	97.4183
372	1114.64	98.0524
373	1116.56	98.7881
374	1118.49	99.6071
375	1120.42	100.417
376	1122.35	101.096
377	1124.28	101.688
378	1126.21	102.205
379	1128.13	102.598
380	1130.06	102.876
381	1131.99	103.03
382	1133.92	103.074
383	1135.85	103.032
384	1137.78	102.897
385	1139.71	102.683
386	1141.63	102.415
387	1143.56	102.127
388	1145.49	101.883
389	1147.42	101.693
390	1149.35	101.506
391	1151.28	101.422
392	1153.2	101.489
393	1155.13	101.547
394	1157.06	101.721
395	1158.99	102.17
396	1160.92	102.687
397	1162.85	103.18
398	1164.77	103.743
399	1166.7	104.393
400	1168.63	105.066
401	1170.56	105.683
402	1172.49	106.273
403	1174.42	106.861
404	1176.35	107.395
405	1178.27	107.864
406	1180.2	108.237
407	1182.13	108.46
408	1184.06	108.555
409	1185.99	108.531
410	1187.92	108.362
411	1189.84	108.071

412	1191.77	107.703
413	1193.7	107.308
414	1195.63	106.937
415	1197.56	106.632
416	1199.49	106.437
417	1201.42	106.385
418	1203.34	106.461
419	1205.27	106.607
420	1207.2	106.789
421	1209.13	106.978
422	1211.06	107.107
423	1212.99	107.16
424	1214.91	107.165
425	1216.84	107.119
426	1218.77	107.004
427	1220.7	106.874
428	1222.63	106.768
429	1224.56	106.625
430	1226.48	106.455
431	1228.41	106.312
432	1230.34	106.183
433	1232.27	106.066
434	1234.2	105.962
435	1236.13	105.85
436	1238.06	105.72
437	1239.98	105.569
438	1241.91	105.398
439	1243.84	105.246
440	1245.77	105.148
441	1247.7	105.049
442	1249.63	104.934
443	1251.55	104.878
444	1253.48	104.892
445	1255.41	104.945
446	1257.34	105.029
447	1259.27	105.089
448	1261.2	105.092
449	1263.13	105.05
450	1265.05	104.929
451	1266.98	104.741
452	1268.91	104.512
453	1270.84	104.251
454	1272.77	103.988
455	1274.7	103.705
456	1276.62	103.393
457	1278.55	103.069
458	1280.48	102.724
459	1282.41	102.396
460	1284.34	102.127
461	1286.27	101.936
462	1288.19	101.862
463	1290.12	101.916

464	1292.05	102.078
465	1293.98	102.348
466	1295.91	102.717
467	1297.84	103.148
468	1299.77	103.59
469	1301.69	103.982
470	1303.62	104.33
471	1305.55	104.639
472	1307.48	104.885
473	1309.41	105.137
474	1311.34	105.385
475	1313.26	105.596
476	1315.19	105.852
477	1317.12	106.109
478	1319.05	106.332
479	1320.98	106.601
480	1322.91	106.841
481	1324.84	106.99
482	1326.76	107.119
483	1328.69	107.225
484	1330.62	107.297
485	1332.55	107.364
486	1334.48	107.392
487	1336.41	107.33
488	1338.33	107.166
489	1340.26	107.102
490	1342.19	107.247
491	1344.12	107.339
492	1346.05	107.303
493	1347.98	107.234
494	1349.9	107.168
495	1351.83	107.14
496	1353.76	107.085
497	1355.69	107.002
498	1357.62	106.945
499	1359.55	106.867
500	1361.48	106.617
501	1363.4	106.42
502	1365.33	106.589
503	1367.26	106.641
504	1369.19	106.559
505	1371.12	106.69
506	1373.05	106.705
507	1374.97	106.771
508	1376.9	107.212
509	1378.83	107.633
510	1380.76	107.929
511	1382.69	108.267
512	1384.62	108.551
513	1386.54	108.6
514	1388.47	108.693
515	1390.4	109.193

516	1392.33	109.527
517	1394.26	109.321
518	1396.19	109.311
519	1398.12	109.447
520	1400.04	109.507
521	1401.97	109.771
522	1403.9	109.712
523	1405.83	109.532
524	1407.76	109.694
525	1409.69	109.742
526	1411.61	109.713
527	1413.54	109.707
528	1415.47	109.678
529	1417.4	109.293
530	1419.33	108.618
531	1421.26	108.769
532	1423.19	108.991
533	1425.11	108.904
534	1427.04	109.033
535	1428.97	108.988
536	1430.9	108.86
537	1432.83	109.284
538	1434.76	109.545
539	1436.68	109.209
540	1438.61	109.607
541	1440.54	110.387
542	1442.47	110.792
543	1444.4	111.184
544	1446.33	111.495
545	1448.25	111.717
546	1450.18	112.208
547	1452.11	112.769
548	1454.04	113.333
549	1455.97	113.226
550	1457.9	112.758
551	1459.83	113.857
552	1461.75	115.042
553	1463.68	115.36
554	1465.61	115.375
555	1467.54	116.057
556	1469.47	116.757
557	1471.4	116.482
558	1473.32	115.895
559	1475.25	116.07
560	1477.18	116.448
561	1479.11	116.76
562	1481.04	116.794
563	1482.97	116.752
564	1484.9	116.698
565	1486.82	116.307
566	1488.75	115.61
567	1490.68	115.078

568	1492.61	115.305
569	1494.54	115.311
570	1496.47	114.261
571	1498.39	113.599
572	1500.32	113.427
573	1502.25	113.379
574	1504.18	113.212
575	1506.11	111.098
576	1508.04	109.534
577	1509.96	110.009
578	1511.89	110.07
579	1513.82	109.931
580	1515.75	109.462
581	1517.68	109.109
582	1519.61	109.176
583	1521.54	108.652
584	1523.46	108.799
585	1525.39	109.309
586	1527.32	109.26
587	1529.25	109.538
588	1531.18	109.672
589	1533.11	108.706
590	1535.03	108.186
591	1536.96	108.437
592	1538.89	107.099
593	1540.82	104.839
594	1542.75	104.504
595	1544.68	104.175
596	1546.61	104.23
597	1548.53	104.279
598	1550.46	104.314
599	1552.39	104.724
600	1554.32	105.209
601	1556.25	105.973
602	1558.18	105.766
603	1560.1	106.02
604	1562.03	108.082
605	1563.96	109.396
606	1565.89	110.181
607	1567.82	110.876
608	1569.75	110.956
609	1571.67	111.71
610	1573.6	112.783
611	1575.53	112.592
612	1577.46	112.557
613	1579.39	113.502
614	1581.32	114.035
615	1583.25	114.215
616	1585.17	114.424
617	1587.1	114.55
618	1589.03	114.582
619	1590.96	114.594

620	1592.89	114.567
621	1594.82	114.418
622	1596.74	114.183
623	1598.67	113.97
624	1600.6	113.726
625	1602.53	113.461
626	1604.46	113.306
627	1606.39	113.134
628	1608.32	112.917
629	1610.24	112.725
630	1612.17	112.75
631	1614.1	112.949
632	1616.03	112.373
633	1617.96	111.864
634	1619.89	112.439
635	1621.81	112.399
636	1623.74	111.715
637	1625.67	111.665
638	1627.6	111.506
639	1629.53	111.167
640	1631.46	111.3
641	1633.38	111.198
642	1635.31	109.925
643	1637.24	109.454
644	1639.17	110.113
645	1641.1	110.367
646	1643.03	110.529
647	1644.96	110.406
648	1646.88	109.169
649	1648.81	109.457
650	1650.74	110.37
651	1652.67	108.677
652	1654.6	107.676
653	1656.53	108.936
654	1658.45	109.47
655	1660.38	109.408
656	1662.31	108.967
657	1664.24	108.772
658	1666.17	109.27
659	1668.1	109.059
660	1670.02	108.28
661	1671.95	108.737
662	1673.88	108.952
663	1675.81	108.549
664	1677.74	108.818
665	1679.67	109.201
666	1681.6	109.381
667	1683.52	108.645
668	1685.45	108.034
669	1687.38	109.011
670	1689.31	109.373
671	1691.24	109.621

672	1693.17	110.267
673	1695.09	109.739
674	1697.02	109.612
675	1698.95	109.907
676	1700.88	109.327
677	1702.81	110.195
678	1704.74	110.966
679	1706.67	111.138
680	1708.59	111.872
681	1710.52	112.613
682	1712.45	113.096
683	1714.38	113.499
684	1716.31	113.467
685	1718.24	113.474
686	1720.16	114.59
687	1722.09	115.708
688	1724.02	116.296
689	1725.95	117.073
690	1727.88	117.687
691	1729.81	118.21
692	1731.73	118.941
693	1733.66	118.696
694	1735.59	119.172
695	1737.52	120.553
696	1739.45	121.034
697	1741.38	121.254
698	1743.31	121.881
699	1745.23	122.289
700	1747.16	122.538
701	1749.09	122.578
702	1751.02	122.427
703	1752.95	122.963
704	1754.88	123.53
705	1756.8	123.47
706	1758.73	123.785
707	1760.66	123.972
708	1762.59	123.772
709	1764.52	124.335
710	1766.45	124.629
711	1768.38	124.541
712	1770.3	124.664
713	1772.23	124.289
714	1774.16	124.393
715	1776.09	125.094
716	1778.02	125.39
717	1779.95	125.359
718	1781.87	125.473
719	1783.8	125.641
720	1785.73	125.636
721	1787.66	125.889
722	1789.59	126.138
723	1791.52	125.658

724	1793.44	125.404
725	1795.37	125.873
726	1797.3	126.188
727	1799.23	126.092
728	1801.16	125.927
729	1803.09	126.087
730	1805.02	126.328
731	1806.94	126.422
732	1808.87	126.388
733	1810.8	126.249
734	1812.73	126.331
735	1814.66	126.507
736	1816.59	126.524
737	1818.51	126.478
738	1820.44	126.537
739	1822.37	126.596
740	1824.3	126.311
741	1826.23	126.277
742	1828.16	126.369
743	1830.09	126.001
744	1832.01	126.135
745	1833.94	126.521
746	1835.87	126.532
747	1837.8	126.471
748	1839.73	126.618
749	1841.66	126.751
750	1843.58	126.236
751	1845.51	125.926
752	1847.44	126.333
753	1849.37	126.542
754	1851.3	126.623
755	1853.23	126.679
756	1855.15	126.674
757	1857.08	126.718
758	1859.01	126.703
759	1860.94	126.724
760	1862.87	126.778
761	1864.8	126.854
762	1866.73	126.749
763	1868.65	126.325
764	1870.58	126.354
765	1872.51	126.747
766	1874.44	126.86
767	1876.37	126.839
768	1878.3	126.842
769	1880.22	126.841
770	1882.15	126.848
771	1884.08	126.802
772	1886.01	126.865
773	1887.94	126.816
774	1889.87	126.583
775	1891.8	126.693

776	1893.72	126.808
777	1895.65	126.715
778	1897.58	126.755
779	1899.51	126.802
780	1901.44	126.781
781	1903.37	126.768
782	1905.29	126.778
783	1907.22	126.726
784	1909.15	126.622
785	1911.08	126.601
786	1913.01	126.684
787	1914.94	126.807
788	1916.86	126.637
789	1918.79	126.439
790	1920.72	126.584
791	1922.65	126.564
792	1924.58	126.542
793	1926.51	126.711
794	1928.44	126.758
795	1930.36	126.766
796	1932.29	126.76
797	1934.22	126.744
798	1936.15	126.755
799	1938.08	126.815
800	1940.01	126.85
801	1941.93	126.661
802	1943.86	126.579
803	1945.79	126.728
804	1947.72	126.799
805	1949.65	126.84
806	1951.58	126.887
807	1953.5	126.879
808	1955.43	126.857
809	1957.36	126.872
810	1959.29	126.9
811	1961.22	126.873
812	1963.15	126.881
813	1965.08	126.875
814	1967	126.768
815	1968.93	126.758
816	1970.86	126.822
817	1972.79	126.817
818	1974.72	126.814
819	1976.65	126.809
820	1978.57	126.789
821	1980.5	126.763
822	1982.43	126.746
823	1984.36	126.768
824	1986.29	126.742
825	1988.22	126.71
826	1990.15	126.657
827	1992.07	126.531

828	1994	126.563
829	1995.93	126.679
830	1997.86	126.658
831	1999.79	126.614
832	2001.72	126.622
833	2003.64	126.643
834	2005.57	126.634
835	2007.5	126.599
836	2009.43	126.596
837	2011.36	126.6
838	2013.29	126.604
839	2015.21	126.585
840	2017.14	126.522
841	2019.07	126.537
842	2021	126.614
843	2022.93	126.639
844	2024.86	126.638
845	2026.79	126.646
846	2028.71	126.653
847	2030.64	126.651
848	2032.57	126.646
849	2034.5	126.651
850	2036.43	126.658
851	2038.36	126.664
852	2040.28	126.638
853	2042.21	126.615
854	2044.14	126.649
855	2046.07	126.667
856	2048	126.657
857	2049.93	126.665
858	2051.86	126.675
859	2053.78	126.676
860	2055.71	126.677
861	2057.64	126.678
862	2059.57	126.663
863	2061.5	126.642
864	2063.43	126.613
865	2065.35	126.58
866	2067.28	126.588
867	2069.21	126.614
868	2071.14	126.62
869	2073.07	126.611
870	2075	126.588
871	2076.92	126.572
872	2078.85	126.566
873	2080.78	126.566
874	2082.71	126.561
875	2084.64	126.54
876	2086.57	126.526
877	2088.5	126.519
878	2090.42	126.509
879	2092.35	126.5

880	2094.28	126.499
881	2096.21	126.512
882	2098.14	126.527
883	2100.07	126.543
884	2101.99	126.554
885	2103.92	126.551
886	2105.85	126.553
887	2107.78	126.558
888	2109.71	126.565
889	2111.64	126.58
890	2113.57	126.59
891	2115.49	126.6
892	2117.42	126.602
893	2119.35	126.605
894	2121.28	126.615
895	2123.21	126.611
896	2125.14	126.616
897	2127.06	126.637
898	2128.99	126.635
899	2130.92	126.628
900	2132.85	126.642
901	2134.78	126.652
902	2136.71	126.653
903	2138.63	126.656
904	2140.56	126.661
905	2142.49	126.663
906	2144.42	126.662
907	2146.35	126.66
908	2148.28	126.652
909	2150.21	126.651
910	2152.13	126.652
911	2154.06	126.646
912	2155.99	126.634
913	2157.92	126.628
914	2159.85	126.634
915	2161.78	126.627
916	2163.7	126.604
917	2165.63	126.603
918	2167.56	126.604
919	2169.49	126.593
920	2171.42	126.597
921	2173.35	126.603
922	2175.28	126.598
923	2177.2	126.592
924	2179.13	126.58
925	2181.06	126.576
926	2182.99	126.586
927	2184.92	126.596
928	2186.85	126.6
929	2188.77	126.605
930	2190.7	126.618
931	2192.63	126.624

932	2194.56	126.621
933	2196.49	126.628
934	2198.42	126.639
935	2200.34	126.64
936	2202.27	126.651
937	2204.2	126.675
938	2206.13	126.685
939	2208.06	126.678
940	2209.99	126.675
941	2211.92	126.692
942	2213.84	126.706
943	2215.77	126.686
944	2217.7	126.671
945	2219.63	126.69
946	2221.56	126.708
947	2223.49	126.714
948	2225.41	126.717
949	2227.34	126.711
950	2229.27	126.707
951	2231.2	126.715
952	2233.13	126.726
953	2235.06	126.725
954	2236.98	126.711
955	2238.91	126.704
956	2240.84	126.7
957	2242.77	126.692
958	2244.7	126.683
959	2246.63	126.681
960	2248.56	126.681
961	2250.48	126.665
962	2252.41	126.652
963	2254.34	126.663
964	2256.27	126.669
965	2258.2	126.669
966	2260.13	126.671
967	2262.05	126.655
968	2263.98	126.629
969	2265.91	126.618
970	2267.84	126.617
971	2269.77	126.6
972	2271.7	126.582
973	2273.63	126.592
974	2275.55	126.602
975	2277.48	126.596
976	2279.41	126.595
977	2281.34	126.607
978	2283.27	126.624
979	2285.2	126.617
980	2287.12	126.597
981	2289.05	126.584
982	2290.98	126.566
983	2292.91	126.545

984	2294.84	126.505
985	2296.77	126.461
986	2298.69	126.44
987	2300.62	126.422
988	2302.55	126.42
989	2304.48	126.427
990	2306.41	126.391
991	2308.34	126.315
992	2310.27	126.227
993	2312.19	126.124
994	2314.12	126.098
995	2316.05	126.176
996	2317.98	126.174
997	2319.91	126.051
998	2321.84	125.89
999	2323.76	125.772
1000	2325.69	125.797
1001	2327.62	125.859
1002	2329.55	125.723
1003	2331.48	125.533
1004	2333.41	125.569
1005	2335.34	125.573
1006	2337.26	125.563
1007	2339.19	125.527
1008	2341.12	125.295
1009	2343.05	125.037
1010	2344.98	124.414
1011	2346.91	124.656
1012	2348.83	125.934
1013	2350.76	126.766
1014	2352.69	126.486
1015	2354.62	126.021
1016	2356.55	125.577
1017	2358.48	125.2
1018	2360.4	124.96
1019	2362.33	124.609
1020	2364.26	124.481
1021	2366.19	124.418
1022	2368.12	124.361
1023	2370.05	124.448
1024	2371.98	124.577
1025	2373.9	124.681
1026	2375.83	124.85
1027	2377.76	125.055
1028	2379.69	125.262
1029	2381.62	125.521
1030	2383.55	125.78
1031	2385.47	125.981
1032	2387.4	126.127
1033	2389.33	126.204
1034	2391.26	126.209
1035	2393.19	126.191

1036	2395.12	126.175
1037	2397.05	126.158
1038	2398.97	126.153
1039	2400.9	126.154
1040	2402.83	126.151
1041	2404.76	126.125
1042	2406.69	126.089
1043	2408.62	126.096
1044	2410.54	126.106
1045	2412.47	126.08
1046	2414.4	126.058
1047	2416.33	126.058
1048	2418.26	126.054
1049	2420.19	126.033
1050	2422.11	126.025
1051	2424.04	126.032
1052	2425.97	126.016
1053	2427.9	125.993
1054	2429.83	125.978
1055	2431.76	125.946
1056	2433.69	125.925
1057	2435.61	125.942
1058	2437.54	125.947
1059	2439.47	125.919
1060	2441.4	125.885
1061	2443.33	125.862
1062	2445.26	125.852
1063	2447.18	125.846
1064	2449.11	125.834
1065	2451.04	125.807
1066	2452.97	125.779
1067	2454.9	125.767
1068	2456.83	125.749
1069	2458.76	125.721
1070	2460.68	125.692
1071	2462.61	125.66
1072	2464.54	125.631
1073	2466.47	125.615
1074	2468.4	125.602
1075	2470.33	125.573
1076	2472.25	125.553
1077	2474.18	125.551
1078	2476.11	125.518
1079	2478.04	125.487
1080	2479.97	125.482
1081	2481.9	125.465
1082	2483.82	125.435
1083	2485.75	125.418
1084	2487.68	125.41
1085	2489.61	125.392
1086	2491.54	125.375
1087	2493.47	125.372

1088	2495.4	125.367
1089	2497.32	125.356
1090	2499.25	125.352
1091	2501.18	125.349
1092	2503.11	125.333
1093	2505.04	125.306
1094	2506.97	125.29
1095	2508.89	125.301
1096	2510.82	125.296
1097	2512.75	125.263
1098	2514.68	125.265
1099	2516.61	125.267
1100	2518.54	125.244
1101	2520.46	125.241
1102	2522.39	125.24
1103	2524.32	125.233
1104	2526.25	125.228
1105	2528.18	125.207
1106	2530.11	125.184
1107	2532.04	125.175
1108	2533.96	125.16
1109	2535.89	125.13
1110	2537.82	125.106
1111	2539.75	125.092
1112	2541.68	125.069
1113	2543.61	125.04
1114	2545.53	125.017
1115	2547.46	124.992
1116	2549.39	124.955
1117	2551.32	124.916
1118	2553.25	124.889
1119	2555.18	124.874
1120	2557.11	124.854
1121	2559.03	124.826
1122	2560.96	124.799
1123	2562.89	124.788
1124	2564.82	124.782
1125	2566.75	124.766
1126	2568.68	124.741
1127	2570.6	124.714
1128	2572.53	124.692
1129	2574.46	124.677
1130	2576.39	124.67
1131	2578.32	124.67
1132	2580.25	124.668
1133	2582.17	124.671
1134	2584.1	124.681
1135	2586.03	124.675
1136	2587.96	124.651
1137	2589.89	124.638
1138	2591.82	124.647
1139	2593.75	124.649

1140	2595.67	124.642
1141	2597.6	124.663
1142	2599.53	124.682
1143	2601.46	124.658
1144	2603.39	124.647
1145	2605.32	124.673
1146	2607.24	124.686
1147	2609.17	124.669
1148	2611.1	124.66
1149	2613.03	124.667
1150	2614.96	124.661
1151	2616.89	124.64
1152	2618.82	124.62
1153	2620.74	124.611
1154	2622.67	124.611
1155	2624.6	124.604
1156	2626.53	124.601
1157	2628.46	124.589
1158	2630.39	124.556
1159	2632.31	124.526
1160	2634.24	124.504
1161	2636.17	124.474
1162	2638.1	124.442
1163	2640.03	124.426
1164	2641.96	124.418
1165	2643.88	124.392
1166	2645.81	124.364
1167	2647.74	124.337
1168	2649.67	124.312
1169	2651.6	124.308
1170	2653.53	124.306
1171	2655.46	124.294
1172	2657.38	124.279
1173	2659.31	124.262
1174	2661.24	124.254
1175	2663.17	124.242
1176	2665.1	124.213
1177	2667.03	124.196
1178	2668.95	124.196
1179	2670.88	124.193
1180	2672.81	124.188
1181	2674.74	124.186
1182	2676.67	124.194
1183	2678.6	124.194
1184	2680.53	124.18
1185	2682.45	124.191
1186	2684.38	124.218
1187	2686.31	124.213
1188	2688.24	124.198
1189	2690.17	124.2
1190	2692.1	124.196
1191	2694.02	124.181

1192	2695.95	124.181
1193	2697.88	124.204
1194	2699.81	124.218
1195	2701.74	124.205
1196	2703.67	124.199
1197	2705.59	124.198
1198	2707.52	124.155
1199	2709.45	124.109
1200	2711.38	124.115
1201	2713.31	124.117
1202	2715.24	124.088
1203	2717.17	124.08
1204	2719.09	124.072
1205	2721.02	124.036
1206	2722.95	124.016
1207	2724.88	124.009
1208	2726.81	124
1209	2728.74	123.989
1210	2730.66	123.961
1211	2732.59	123.959
1212	2734.52	123.97
1213	2736.45	123.925
1214	2738.38	123.885
1215	2740.31	123.894
1216	2742.24	123.872
1217	2744.16	123.832
1218	2746.09	123.839
1219	2748.02	123.853
1220	2749.95	123.841
1221	2751.88	123.832
1222	2753.81	123.841
1223	2755.73	123.847
1224	2757.66	123.841
1225	2759.59	123.844
1226	2761.52	123.845
1227	2763.45	123.851
1228	2765.38	123.851
1229	2767.3	123.824
1230	2769.23	123.83
1231	2771.16	123.861
1232	2773.09	123.862
1233	2775.02	123.859
1234	2776.95	123.871
1235	2778.88	123.878
1236	2780.8	123.875
1237	2782.73	123.89
1238	2784.66	123.898
1239	2786.59	123.864
1240	2788.52	123.833
1241	2790.45	123.816
1242	2792.37	123.803
1243	2794.3	123.785

1244	2796.23	123.746
1245	2798.16	123.718
1246	2800.09	123.713
1247	2802.02	123.692
1248	2803.94	123.663
1249	2805.87	123.637
1250	2807.8	123.589
1251	2809.73	123.534
1252	2811.66	123.497
1253	2813.59	123.464
1254	2815.52	123.428
1255	2817.44	123.389
1256	2819.37	123.349
1257	2821.3	123.314
1258	2823.23	123.284
1259	2825.16	123.249
1260	2827.09	123.202
1261	2829.01	123.168
1262	2830.94	123.168
1263	2832.87	123.164
1264	2834.8	123.149
1265	2836.73	123.167
1266	2838.66	123.249
1267	2840.59	123.429
1268	2842.51	123.74
1269	2844.44	124.211
1270	2846.37	124.804
1271	2848.3	125.26
1272	2850.23	125.304
1273	2852.16	124.994
1274	2854.08	124.56
1275	2856.01	124.153
1276	2857.94	123.79
1277	2859.87	123.431
1278	2861.8	123.111
1279	2863.73	122.859
1280	2865.65	122.671
1281	2867.58	122.574
1282	2869.51	122.514
1283	2871.44	122.421
1284	2873.37	122.293
1285	2875.3	122.152
1286	2877.23	122.042
1287	2879.15	121.947
1288	2881.08	121.858
1289	2883.01	121.826
1290	2884.94	121.829
1291	2886.87	121.83
1292	2888.8	121.852
1293	2890.72	121.912
1294	2892.65	121.989
1295	2894.58	122.06

1296	2896.51	122.145
1297	2898.44	122.229
1298	2900.37	122.298
1299	2902.3	122.398
1300	2904.22	122.536
1301	2906.15	122.705
1302	2908.08	122.963
1303	2910.01	123.366
1304	2911.94	123.948
1305	2913.87	124.61
1306	2915.79	125.083
1307	2917.72	125.209
1308	2919.65	125.085
1309	2921.58	124.842
1310	2923.51	124.549
1311	2925.44	124.237
1312	2927.36	123.907
1313	2929.29	123.623
1314	2931.22	123.406
1315	2933.15	123.2
1316	2935.08	123.022
1317	2937.01	122.886
1318	2938.94	122.77
1319	2940.86	122.698
1320	2942.79	122.679
1321	2944.72	122.708
1322	2946.65	122.8
1323	2948.58	122.97
1324	2950.51	123.177
1325	2952.43	123.373
1326	2954.36	123.539
1327	2956.29	123.633
1328	2958.22	123.652
1329	2960.15	123.634
1330	2962.08	123.598
1331	2964.01	123.544
1332	2965.93	123.466
1333	2967.86	123.381
1334	2969.79	123.294
1335	2971.72	123.209
1336	2973.65	123.143
1337	2975.58	123.109
1338	2977.5	123.079
1339	2979.43	123.039
1340	2981.36	123.036
1341	2983.29	123.052
1342	2985.22	123.032
1343	2987.15	122.994
1344	2989.07	122.945
1345	2991	122.895
1346	2992.93	122.857
1347	2994.86	122.802

1348	2996.79	122.752
1349	2998.72	122.735
1350	3000.65	122.715
1351	3002.57	122.67
1352	3004.5	122.615
1353	3006.43	122.562
1354	3008.36	122.484
1355	3010.29	122.367
1356	3012.22	122.277
1357	3014.14	122.226
1358	3016.07	122.146
1359	3018	122.058
1360	3019.93	122.006
1361	3021.86	121.966
1362	3023.79	121.907
1363	3025.72	121.83
1364	3027.64	121.776
1365	3029.57	121.715
1366	3031.5	121.598
1367	3033.43	121.493
1368	3035.36	121.44
1369	3037.29	121.385
1370	3039.21	121.311
1371	3041.14	121.25
1372	3043.07	121.209
1373	3045	121.162
1374	3046.93	121.092
1375	3048.86	120.994
1376	3050.78	120.922
1377	3052.71	120.886
1378	3054.64	120.811
1379	3056.57	120.729
1380	3058.5	120.68
1381	3060.43	120.602
1382	3062.36	120.509
1383	3064.28	120.419
1384	3066.21	120.308
1385	3068.14	120.232
1386	3070.07	120.189
1387	3072	120.119
1388	3073.93	120.038
1389	3075.85	119.927
1390	3077.78	119.804
1391	3079.71	119.722
1392	3081.64	119.662
1393	3083.57	119.6
1394	3085.5	119.536
1395	3087.42	119.471
1396	3089.35	119.404
1397	3091.28	119.356
1398	3093.21	119.311
1399	3095.14	119.219

1400	3097.07	119.129
1401	3099	119.065
1402	3100.92	119.019
1403	3102.85	119.003
1404	3104.78	118.947
1405	3106.71	118.866
1406	3108.64	118.821
1407	3110.57	118.777
1408	3112.49	118.719
1409	3114.42	118.643
1410	3116.35	118.553
1411	3118.28	118.504
1412	3120.21	118.495
1413	3122.14	118.482
1414	3124.07	118.441
1415	3125.99	118.369
1416	3127.92	118.315
1417	3129.85	118.281
1418	3131.78	118.201
1419	3133.71	118.117
1420	3135.64	118.085
1421	3137.56	118.031
1422	3139.49	117.95
1423	3141.42	117.91
1424	3143.35	117.871
1425	3145.28	117.794
1426	3147.21	117.729
1427	3149.13	117.665
1428	3151.06	117.566
1429	3152.99	117.494
1430	3154.92	117.458
1431	3156.85	117.403
1432	3158.78	117.339
1433	3160.71	117.283
1434	3162.63	117.207
1435	3164.56	117.125
1436	3166.49	117.05
1437	3168.42	116.972
1438	3170.35	116.908
1439	3172.28	116.843
1440	3174.2	116.779
1441	3176.13	116.701
1442	3178.06	116.588
1443	3179.99	116.516
1444	3181.92	116.464
1445	3183.85	116.387
1446	3185.78	116.325
1447	3187.7	116.271
1448	3189.63	116.207
1449	3191.56	116.132
1450	3193.49	116.056
1451	3195.42	115.994

1452	3197.35	115.933
1453	3199.27	115.853
1454	3201.2	115.771
1455	3203.13	115.716
1456	3205.06	115.685
1457	3206.99	115.637
1458	3208.92	115.574
1459	3210.84	115.525
1460	3212.77	115.442
1461	3214.7	115.351
1462	3216.63	115.312
1463	3218.56	115.261
1464	3220.49	115.195
1465	3222.42	115.178
1466	3224.34	115.168
1467	3226.27	115.114
1468	3228.2	115.036
1469	3230.13	114.971
1470	3232.06	114.917
1471	3233.99	114.859
1472	3235.91	114.83
1473	3237.84	114.83
1474	3239.77	114.792
1475	3241.7	114.718
1476	3243.63	114.642
1477	3245.56	114.576
1478	3247.49	114.552
1479	3249.41	114.563
1480	3251.34	114.53
1481	3253.27	114.43
1482	3255.2	114.363
1483	3257.13	114.349
1484	3259.06	114.321
1485	3260.98	114.288
1486	3262.91	114.244
1487	3264.84	114.194
1488	3266.77	114.159
1489	3268.7	114.119
1490	3270.63	114.076
1491	3272.55	114.031
1492	3274.48	113.983
1493	3276.41	113.962
1494	3278.34	113.963
1495	3280.27	113.963
1496	3282.2	113.943
1497	3284.13	113.92
1498	3286.05	113.915
1499	3287.98	113.909
1500	3289.91	113.88
1501	3291.84	113.838
1502	3293.77	113.834
1503	3295.7	113.848

1504	3297.62	113.828
1505	3299.55	113.819
1506	3301.48	113.794
1507	3303.41	113.757
1508	3305.34	113.766
1509	3307.27	113.758
1510	3309.2	113.757
1511	3311.12	113.804
1512	3313.05	113.825
1513	3314.98	113.824
1514	3316.91	113.825
1515	3318.84	113.83
1516	3320.77	113.831
1517	3322.69	113.817
1518	3324.62	113.813
1519	3326.55	113.839
1520	3328.48	113.876
1521	3330.41	113.879
1522	3332.34	113.86
1523	3334.26	113.859
1524	3336.19	113.848
1525	3338.12	113.842
1526	3340.05	113.859
1527	3341.98	113.869
1528	3343.91	113.905
1529	3345.84	113.943
1530	3347.76	113.935
1531	3349.69	113.933
1532	3351.62	113.986
1533	3353.55	114.032
1534	3355.48	114.016
1535	3357.41	113.995
1536	3359.33	113.996
1537	3361.26	114.005
1538	3363.19	114.043
1539	3365.12	114.081
1540	3367.05	114.088
1541	3368.98	114.1
1542	3370.9	114.17
1543	3372.83	114.259
1544	3374.76	114.295
1545	3376.69	114.316
1546	3378.62	114.371
1547	3380.55	114.435
1548	3382.48	114.486
1549	3384.4	114.532
1550	3386.33	114.589
1551	3388.26	114.666
1552	3390.19	114.7
1553	3392.12	114.719
1554	3394.05	114.815
1555	3395.97	114.891

1556	3397.9	114.922
1557	3399.83	114.995
1558	3401.76	115.092
1559	3403.69	115.185
1560	3405.62	115.249
1561	3407.55	115.294
1562	3409.47	115.371
1563	3411.4	115.468
1564	3413.33	115.554
1565	3415.26	115.633
1566	3417.19	115.7
1567	3419.12	115.739
1568	3421.04	115.782
1569	3422.97	115.888
1570	3424.9	116.014
1571	3426.83	116.101
1572	3428.76	116.192
1573	3430.69	116.28
1574	3432.61	116.358
1575	3434.54	116.459
1576	3436.47	116.586
1577	3438.4	116.717
1578	3440.33	116.771
1579	3442.26	116.829
1580	3444.19	116.95
1581	3446.11	116.943
1582	3448.04	116.946
1583	3449.97	117.132
1584	3451.9	117.28
1585	3453.83	117.376
1586	3455.76	117.481
1587	3457.68	117.554
1588	3459.61	117.642
1589	3461.54	117.723
1590	3463.47	117.823
1591	3465.4	117.931
1592	3467.33	117.999
1593	3469.26	118.113
1594	3471.18	118.263
1595	3473.11	118.367
1596	3475.04	118.441
1597	3476.97	118.575
1598	3478.9	118.714
1599	3480.83	118.73
1600	3482.75	118.799
1601	3484.68	118.978
1602	3486.61	119.082
1603	3488.54	119.186
1604	3490.47	119.353
1605	3492.4	119.477
1606	3494.32	119.548
1607	3496.25	119.6

1608	3498.18	119.745
1609	3500.11	119.945
1610	3502.04	119.943
1611	3503.97	119.988
1612	3505.9	120.322
1613	3507.82	120.509
1614	3509.75	120.482
1615	3511.68	120.603
1616	3513.61	120.851
1617	3515.54	121.036
1618	3517.47	121.164
1619	3519.39	121.351
1620	3521.32	121.469
1621	3523.25	121.472
1622	3525.18	121.602
1623	3527.11	121.733
1624	3529.04	121.808
1625	3530.97	122.075
1626	3532.89	122.39
1627	3534.82	122.488
1628	3536.75	122.53
1629	3538.68	122.758
1630	3540.61	123.004
1631	3542.54	123.133
1632	3544.46	123.079
1633	3546.39	122.981
1634	3548.32	123.34
1635	3550.25	123.714
1636	3552.18	123.672
1637	3554.11	123.835
1638	3556.03	124.226
1639	3557.96	124.435
1640	3559.89	124.461
1641	3561.82	124.615
1642	3563.75	124.957
1643	3565.68	124.604
1644	3567.61	123.93
1645	3569.53	124.459
1646	3571.46	125.252
1647	3573.39	125.498
1648	3575.32	125.606
1649	3577.25	125.7
1650	3579.18	125.793
1651	3581.1	125.95
1652	3583.03	126.304
1653	3584.96	126.491
1654	3586.89	125.758
1655	3588.82	125.512
1656	3590.75	126.384
1657	3592.68	126.745
1658	3594.6	126.48
1659	3596.53	126.607

1660	3598.46	126.849
1661	3600.39	126.821
1662	3602.32	126.982
1663	3604.25	127.492
1664	3606.17	127.599
1665	3608.1	127.086
1666	3610.03	127.217
1667	3611.96	127.414
1668	3613.89	127.299
1669	3615.82	127.898
1670	3617.74	127.749
1671	3619.67	127.092
1672	3621.6	127.624
1673	3623.53	128.35
1674	3625.46	129.186
1675	3627.39	128.568
1676	3629.32	126.744
1677	3631.24	127.538
1678	3633.17	128.418
1679	3635.1	128.448
1680	3637.03	128.783
1681	3638.96	129.008
1682	3640.89	129.032
1683	3642.81	129.403
1684	3644.74	129.863
1685	3646.67	129.655
1686	3648.6	128.145
1687	3650.53	127.278
1688	3652.46	128.787
1689	3654.38	129.77
1690	3656.31	129.03
1691	3658.24	129.209
1692	3660.17	129.914
1693	3662.1	130.073
1694	3664.03	130.21
1695	3665.96	130.813
1696	3667.88	130.803
1697	3669.81	129.249
1698	3671.74	129.938
1699	3673.67	130.762
1700	3675.6	128.758
1701	3677.53	128.907
1702	3679.45	130.024
1703	3681.38	130.209
1704	3683.31	130.696
1705	3685.24	131.278
1706	3687.17	130.819
1707	3689.1	129.353
1708	3691.03	129.141
1709	3692.95	129.977
1710	3694.88	130.427
1711	3696.81	130.6

1712	3698.74	130.88
1713	3700.67	130.486
1714	3702.6	129.875
1715	3704.52	130.393
1716	3706.45	130.865
1717	3708.38	130.74
1718	3710.31	129.81
1719	3712.24	128.781
1720	3714.17	129.541
1721	3716.09	130.395
1722	3718.02	130.486
1723	3719.95	130.294
1724	3721.88	130.035
1725	3723.81	129.975
1726	3725.74	129.637
1727	3727.67	129.865
1728	3729.59	130.604
1729	3731.52	130.349
1730	3733.45	129.396
1731	3735.38	128.65
1732	3737.31	128.466
1733	3739.24	129.514
1734	3741.16	131.056
1735	3743.09	130.184
1736	3745.02	128.14
1737	3746.95	129.636
1738	3748.88	130.18
1739	3750.81	128.236
1740	3752.74	128.305
1741	3754.66	129.42
1742	3756.59	130.059
1743	3758.52	129.586
1744	3760.45	129.417
1745	3762.38	130.244
1746	3764.31	130.162
1747	3766.23	130.014
1748	3768.16	130.048
1749	3770.09	129.422
1750	3772.02	129.636
1751	3773.95	130.227
1752	3775.88	130.489
1753	3777.8	130.197
1754	3779.73	129.597
1755	3781.66	129.921
1756	3783.59	130.179
1757	3785.52	129.879
1758	3787.45	130.104
1759	3789.38	130.265
1760	3791.3	130.259
1761	3793.23	130.687
1762	3795.16	130.197
1763	3797.09	129.975

1764	3799.02	130.74
1765	3800.95	129.266
1766	3802.87	129.019
1767	3804.8	130.255
1768	3806.73	129.359
1769	3808.66	129.203
1770	3810.59	130.162
1771	3812.52	130.976
1772	3814.45	130.543
1773	3816.37	129.285
1774	3818.3	130.172
1775	3820.23	129.51
1776	3822.16	128.37
1777	3824.09	129.678
1778	3826.02	129.832
1779	3827.94	129.847
1780	3829.87	130.315
1781	3831.8	129.888
1782	3833.73	130.06
1783	3835.66	130.182
1784	3837.59	129.058
1785	3839.51	128.734
1786	3841.44	129.219
1787	3843.37	129.317
1788	3845.3	129.657
1789	3847.23	130.302
1790	3849.16	131.067
1791	3851.09	131.301
1792	3853.01	128.52
1793	3854.94	127.342
1794	3856.87	129.128
1795	3858.8	130.057
1796	3860.73	130.282
1797	3862.66	129.936
1798	3864.58	129.252
1799	3866.51	130.104
1800	3868.44	130.048
1801	3870.37	128.916
1802	3872.3	129.434
1803	3874.23	129.391
1804	3876.16	129.833
1805	3878.08	130.609
1806	3880.01	129.504
1807	3881.94	129.76
1808	3883.87	130.192
1809	3885.8	129.031
1810	3887.73	129.797
1811	3889.65	130.372
1812	3891.58	129.299
1813	3893.51	129.502
1814	3895.44	130.436
1815	3897.37	130.496

1816	3899.3	129.874
1817	3901.22	129.532
1818	3903.15	129.133
1819	3905.08	128.867
1820	3907.01	129.534
1821	3908.94	130.08
1822	3910.87	130.115
1823	3912.8	130.317
1824	3914.72	130.348
1825	3916.65	129.908
1826	3918.58	129.619
1827	3920.51	129.831
1828	3922.44	129.996
1829	3924.37	129.756
1830	3926.29	129.774
1831	3928.22	130.109
1832	3930.15	129.964
1833	3932.08	129.539
1834	3934.01	129.68
1835	3935.94	129.986
1836	3937.86	130.09
1837	3939.79	130.178
1838	3941.72	129.912
1839	3943.65	129.736
1840	3945.58	130.014
1841	3947.51	129.959
1842	3949.44	129.766
1843	3951.36	129.875
1844	3953.29	130.009
1845	3955.22	130.058
1846	3957.15	130.071
1847	3959.08	130.103
1848	3961.01	130.051
1849	3962.93	130.014
1850	3964.86	130.11
1851	3966.79	130.14
1852	3968.72	130.095
1853	3970.65	130.077
1854	3972.58	130.089
1855	3974.51	130.095
1856	3976.43	130.111
1857	3978.36	130.154
1858	3980.29	130.15
1859	3982.22	130.128
1860	3984.15	130.138
1861	3986.08	130.166
1862	3988	130.173
1863	3989.93	130.134
1864	3991.86	130.109
1865	3993.79	130.09
1866	3995.72	130.08
1867	3997.65	130.121

1868	3999.57	130.139
1869	4001.5	130.117
